# Entropy production rate is maximized in non-contractile actomyosin

**DOI:** 10.1038/s41467-018-07413-5

**Published:** 2018-11-23

**Authors:** Daniel S. Seara, Vikrant Yadav, Ian Linsmeier, A. Pasha Tabatabai, Patrick W. Oakes, S. M. Ali Tabei, Shiladitya Banerjee, Michael P. Murrell

**Affiliations:** 1 Department of Physics, Yale University, 217 Prospect Street, New Haven, CT 06511 USA; 2 Systems Biology Institute, Yale University, 850 West Campus Drive, West Haven, CT 06516 USA; 3 Department of Biomedical Engineering, Yale University, 55 Prospect Street, New Haven, CT 06511 USA; 4 Department of Physics & Astronomy, and Department of Biology, University of Rochester, Rochester, NY 14627 USA; 5 Physics Department, University of Northern Iowa, Cedar Falls, IA 50614 USA; 6 Department of Physics and Astronomy, Institute for the Physics of Living Systems, University College London, Gower Street, London, WC1E 6BT UK

## Abstract

The actin cytoskeleton is an active semi-flexible polymer network whose non-equilibrium properties coordinate both stable and contractile behaviors to maintain or change cell shape. While myosin motors drive the actin cytoskeleton out-of-equilibrium, the role of myosin-driven active stresses in the accumulation and dissipation of mechanical energy is unclear. To investigate this, we synthesize an actomyosin material in vitro whose active stress content can tune the network from stable to contractile. Each increment in activity determines a characteristic spectrum of actin filament fluctuations which is used to calculate the total mechanical work and the production of entropy in the material. We find that the balance of work and entropy does not increase monotonically and the entropy production rate is maximized in the non-contractile, stable state of actomyosin. Our study provides evidence that the origins of entropy production and activity-dependent dissipation relate to disorder in the molecular interactions between actin and myosin.

## Abstract (web summary)

Myosin motors drive the actin cytoskeleton out-of-equilibrium, but the role of myosin-driven active stresses in the accumulation and dissipation of mechanical work is unclear. Here, the authors synthesize an actomyosin material and find that the rate of entropy production increases non-monotonically with increasing accumulation of active stresses.

## Introduction

The eukaryotic cytoskeleton is an active, viscoelastic material that exhibits a wide range of dynamic responses to both its internal and external environment^1^. This includes polarizing contractile flows during embryonic development^2^ and cell division in the adult^3,4^. By contrast, there are dynamic steady states, including ratcheting motions in the *Drosophila* wing^5^, excitable wave motion in the *Xenopus* oocyte^6^ and active nematic fluctuations in the mitotic spindle^7^. It is generally accepted that the driving force for many of these processes originate from both filament turnover and the relative sliding between molecular motors and cytoskeletal polymers along their long axis^8–10^. For example, in vitro, rigid microtubule filaments^11,12^ reach a flowing dynamic steady state under the influence of kinesin motors^12,13^. As a consequence, microtubule networks retain their overall density and architecture^7^ or yield to extensile flows^14^. By contrast, myosin motor activity on semi-flexible filamentous (F) actin leads to filament buckling^15^ and severing at high curvatures^16,17^. As a result, F-actin networks experience macroscopic architectural changes^18^ and large strains^15^ during destabilizing contractile flows^19^. Thus, it remains unclear how networks of semi-flexible polymers can maintain a dynamic steady state in the presence of active stress. More generally, the relationship between the out-of-equilibrium accumulation and dissipation of mechanical stresses and the stabilization of active materials is unknown.

In this work, we characterize the thermodynamic criteria for the maintenance of dynamic stability in an active biomimetic material composed of semi-flexible F-actin through determination of the rate of entropy production as a function of molecular motor activity. First, we systematically identify the range of motor activity that differentiates macroscopic contractility (unstable) from steady-state non-contractile behavior (stable). Next, we determine the effect of activity on the microscopic balance of mechanical work and the production of entropy from the myosin-induced bending of individual F-actin. This provides a quantitative relationship between how far the system is from equilibrium with its propensity to dissipate mechanical energy. We then correlate network and filament properties to associate the accumulation of mechanical work and the production of entropy with the mechanical stability of the bulk material. Finally, we compute the entropy produced in the actin network in time and per individual myosin filament and correlate the motions of myosin filaments with the bulk dissipation that stabilizes the material.

## Results

### F-actin self-assembles into a 2D nematically ordered network

**Fig. 1:**
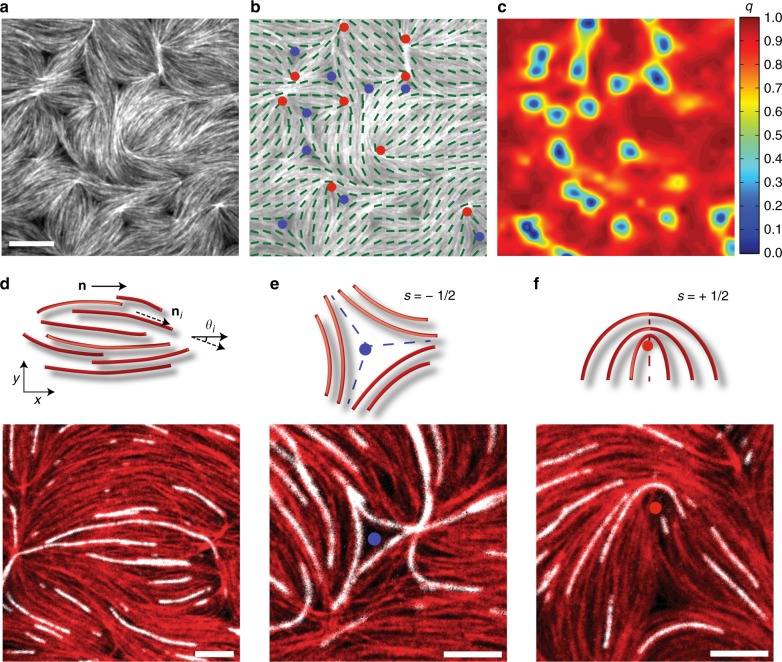
Synthesis of a 2D actomyosin network with nematic ordering. **a** Fluorescent F-actin crowded to the bilayer. Scale bar is 10μm. **b** Location of disclinations with topological defect charges *s*=−1/2 and *s*=+1/2, shown by blue and red dots respectively. Green lines represent nematic director of the F-actin gel. **c** Heat map of the nematic order parameter $q = 2\langle \cos ^2\theta - 1/2\rangle$. **d** (top) Schematic of a nematically ordered domain comprised of many actin filaments, where $\textbf n$ is the nematic director of the entire domain, **n**_*i*_ is the local alignment of a single F-actin, and *θ*_*i*_ is the angle between them and (bottom) image of a single nematic domain. Both red and white show actin filaments labeled with different fluorophores, polymerized separately, and combined at a 1:50 ratio prior to crowding to visualize individual filaments within the larger network. **e** Schematic (top) and image (bottom) of a −1/2 disclination defect and local nematic ordering in quasi 2D F-actin. **f** Schematic (top) and image (bottom) of a +1/2 disclination topological defect and local nematic ordering in quasi 2D F-actin network. Scale bars in **d**–**f** are 5 μm

F-actin is crowded to the surface of a phospholipid bilayer over time due to the depletion forces induced by methylcellulose (0.25% MC)^20^ (Supplementary Movie 1). In the absence of adhesion between actin filaments and membrane, the filaments change their spatial orientation to establish a net direction upon reaching the membrane surface. This reorganization of the network generates local domains of nematic alignment, quantified by the coarse-grained nematic order parameter $q = 2\langle \cos ^2\theta - 1/2\rangle$ (Fig. 1a–d, Methods, Supplementary Figure 1, Supplementary Methods). As F-actin accumulates on the bilayer, the network transitions continuously from an isotropic to a nematic phase (Supplementary Figure 1, 2). The nematic domains originate from and terminate in regions of disorganized F-actin containing disclination defects with topological charge ±1/2^21^. −1/2 defects are formed by moderate F-actin bending in radial directions around a central void, whereas +1/2 defects form due to highly bent F-actin oriented along a single direction about a central core (Fig. 1e, f).

While the F-actin network exhibits the same defects and symmetries as a traditional nematic liquid crystal composed of short, rigid rods^12,22^, the average F-actin length in our experiments is ~10 μm, comparable to their persistence length^23^. As such, defects form due to bending and entanglement of a small number of individual actin filaments (Fig. 1e, f, bottom). Further, we do not observe defect motion or annihilation, reported in other biopolymer nematic liquid crystals^12,24,25^, thus the effect of myosin activity on network architecture and stability is unclear.

### Myosin activity alters nematic order in F-actin networks

Thus far, the dynamics of myosin-driven F-actin networks have largely been attributed to contractile flow^15,16,18,19,26–30^. We have previously shown that contractile flow occurs in a cooperative manner above a critical myosin thick filament density, *ρ*_c_^16,31^. Thus, our first metric for motor “activity” is one that is dependent solely on myosin density and results in contractile stresses, *ζ*(*ρ*). For *ρ* > *ρ*_c_, filament buckling coincides with network contraction as it shortens the filament end-to-end length^32,33^. However, the impact of sub-contractile densities of myosin (*ρ* < *ρ*_c_) on the dynamics of F-actin is unclear. To this end, we accumulate myosin-thick filaments on the assembled F-actin network at densities above and below *ρ*_c_ to compare and contrast the impact of activity on actomyosin network assembly dynamics.

**Fig. 2:**
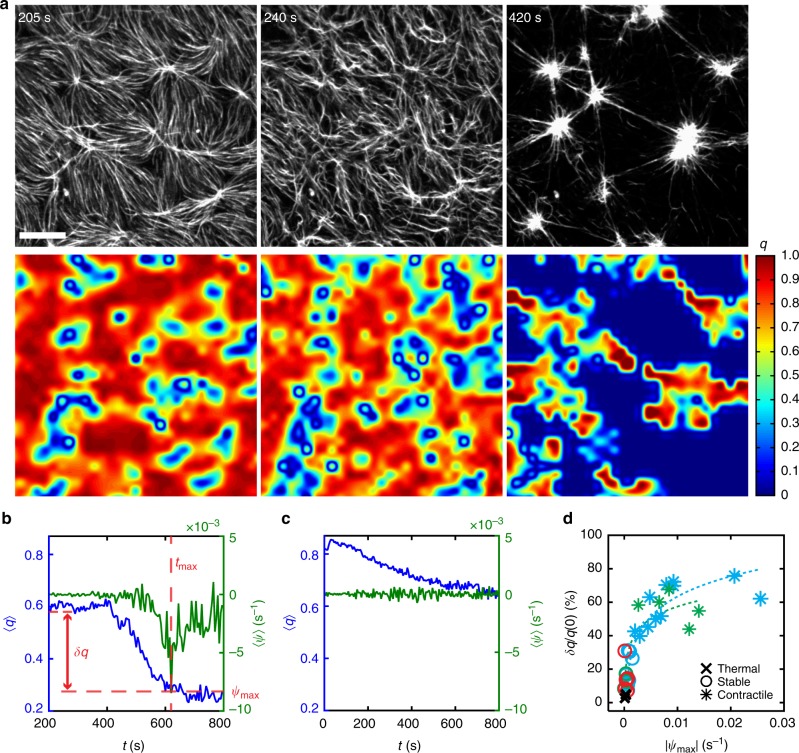
Myosin density affects F-actin nematic order in both contractile and stable states. **a** (top) Fluorescent F-actin network undergoing contraction. Scale bar is 10μm. (bottom) Heat map of scalar nematic order parameter *q*. Myosin dimer added at *t* *=* 0. **b** Spatially averaged F-actin nematic order parameter measured (*q*, blue) and divergence of F-actin velocity (*ψ*, green) 200 s after the onset of myosin addition when *ρ* > *ρ*_c_. Time of maximum magnitude of divergence (*t*_max_) indicated by vertical dotted red line. Difference between nematic order at *t* = 0 and at time of maximum divergence (*δq* = *q*(0) – *q*(*t*_max_)) indicated by horizontal dotted red line. **c** Spatially averaged nematic order (blue) and divergence of the velocity (green) for a stable actomyosin network, *ρ* < *ρ*_c_, where myosin is added at *t* = 0. **d** Percent change in nematic order (δ*q*/*q*(0)) for thermal (cross), stable (circle), and contractile (star) network states. The marker color denotes the myosin isoform added to each experiment (skeletal muscle myosin (SkMM) = blue, smooth muscle myosin (SmMM) = green, non-muscle myosin (NMM) = red, no myosin = black). We define non-contractile, ‘stable’ (S) networks as those with *ψ*_max_ < *ψ*_c_ = 2 x10^−3 ^s^−1^, contractile networks (C) for *ψ*_max_ > *ψ*_c_, and networks for which no myosin added as ‘thermal’ networks (T)

To assemble the actomyosin network, myosin dimers are added at *t* = 0, accumulating and forming thick filament assemblies within ~100 s. After the addition of myosin, we calculate the divergence of the F-actin velocity field, which yields the macroscopic strain rate, $\psi \left( t \right) = \langle \nabla \cdot \textbf v\left( t \right)\rangle$. We also calculate the spatially averaged nematic order of the F-actin network, 〈*q*(*t*)〉 (Fig. 2a, b, Supplementary Figure 3, 4) and the change in the average nematic order *δ*〈*q*(*t*)〉, defined as the difference between the nematic order prior to the addition of myosin to the nematic order at time *t*. For *ρ* > *ρ*_c_, the strain rate decreases during contraction until it reaches a maximum in its magnitude, *ψ*_max_. Relatedly, the change in the nematic order also increases with time, representing a loss in F-actin alignment due to myosin activity. However, the loss of 〈*q*(*t*)〉 precedes the drop in *ψ*(*t*) in time (Supplementary Movie 2), suggesting there may be dynamics of actomyosin (*δq* *≠* 0) that are non-contractile (*ψ* ~ 0).

Indeed, for *ρ*  < *ρ*_c_, *ψ*_max_ decays to zero although *δq* does not—the network retains up to 70% of its original nematic order (Fig. 2c, d). This state (*ψ* = 0) is established quickly and can be experimentally observed for up to 1 h (Supplementary Movie 3). Thus, myosin activity can drive the establishment of a steady-state defined by changes in F-actin structural dynamics absent of contractile flows. To this end, we characterize the dynamics of this steady state by analyzing the fluctuations in the nematic alignment of the F-actin network using both experiment and simulation.

### Density fluctuations are elevated in non-contractile state

**Fig. 3:**
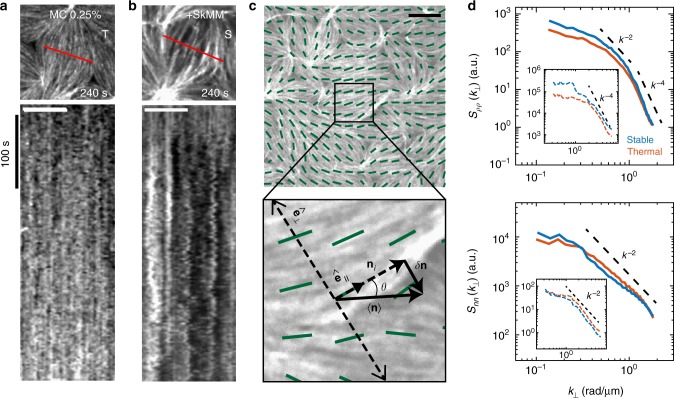
Intermediate activity enhances fluctuations in stable actomyosin. **a**, **b** Fluorescent F-actin kymograph across a single nematic domain of a 2D network in the presence of 0.25% methylcellulose (MC) (T, **a**), and 0.25% MC + SkMM (S, **b**). White scale bar is 5 μm. **c** 2D F-actin network in the presence of 0.25% MC with alignment vector field (green), scaled by the local nematic order parameter, overlaid. Lower panel shows a schematic of the local alignment vector (**n**_*i*_), local coordinate system defined by the axes parallel $\left( {{\hat{\mathbf e}}_\parallel } \right)$ and perpendicular $\left( {{\hat{\mathbf e}}_ \bot } \right)$ to the local alignment vector, and local alignment vector fluctuations $\left( {\delta {\mathbf{n}}_i \equiv {\mathbf{n}}_i - \langle {\mathbf{n}}_i\rangle } \right)$ used to calculate fluctuation autocorrelations. Scale bar is 10 μm. **d** Equal time spatial density-density (*S*_*ρρ*_, top) and director-director (*S*_*nn*_, bottom) fluctuation autocorrelations as a function of the perpendicular wave vector (*k*_⊥_). The spatial autocorrelations are shown for thermal (orange, 0.25% MC, *N* = 16) and stable (blue, SkMM, *N* = 6) experimental conditions within the F-actin network. All experiments contain the same concentration of F-actin (2.32 μM) with 0.25% MC. The dashed black lines follow the predicted autocorrelation decay by active nematic liquid crystal theory. Insets show corresponding results for *S*_*ρρ*_ and *S*_*nn*_ from agent-based simulation. Each curve is an average over 3 simulations. Mean length of simulated filaments is 4.3 μm

Kymographs of F-actin fluorescence intensity exhibit distinct fluctuations in space for thermal (T) and stable networks (S) (Fig. 3a, b). To quantify these fluctuations, we measure the Fourier transform of the equal-time spatial autocorrelation function of the F-actin density fluctuations (*S*_*ρρ*_) and the nematic director fluctuations (*S*_*nn*_) perpendicular to the local axis of F-actin alignment as a function of wavenumber *k*_⊥_ (Fig. 3c, d, Supplementary Note 1, Supplementary Figure 5).

The addition of myosin motors (*ρ*  < *ρ*_c_*; S*) increases the magnitude of the fluctuation present as an elevated *S*_*ρρ*_ at low *k*_⊥_, consistent with the qualitatively larger fluctuations in F-actin fluorescence intensity in kymographs (Fig. 3a, b). Using a model for active nematic gels, this may be indicative of an increase in filament mobility or orientational noise (Supplementary Note 1). While the active gel model fits for short, rigid filaments ($\bar l \approx$ 2.5 μm, *N*_fils_ = 200), we find deviations from this model for long filaments at high *k*_⊥_, suggesting the relationship between activity and filament fluctuations is complex (Supplementary Figure 6). Using agent-based simulations^34^, we show that this deviation at high *k*_⊥_ arises from length dependent but activity-independent dissipative effects (Supplementary Note 2, Supplementary Figure 7).

Therefore, a uniform active stress and simple viscosity may not fully capture the dependence of myosin activity on F-actin fluctuations and dissipation. To this end, we investigate the role of motor activity on dissipation on the filament scale by using a model-free estimate of the rate of entropy production.

### Activity-dependent dissipation is maximized in stable state

When driven out of equilibrium, microscopic systems obey fluctuation theorems that relate the irreversibility of a process to the amount of entropy produced in that process^35–37^. Myosin motors operate far from equilibrium by hydrolyzing ATP to generate forces on F-actin, as previously quantified by deviations from the fluctuation dissipation theorem^38^. Here we show that the dissipated energy in actomyosin is amenable to quantification using the framework of stochastic thermodynamics^37^.

**Fig. 4:**
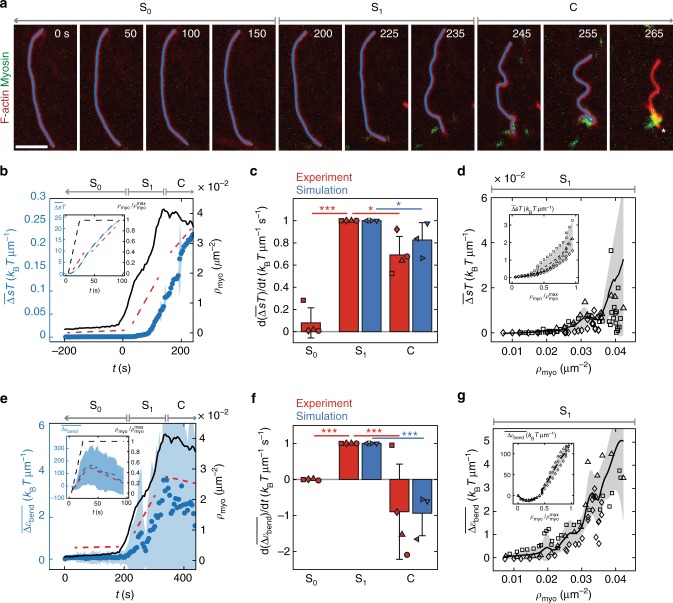
Entropy production rate is highest in non-contractile (stable) actomyosin. **a** Example of experiment with 1% labeled filaments (red) as myosin accumulates (green). Filaments are tracked (blue line) until a severing event, indicated by white asterisk. Gray arrows indicate three states of entropy production rate: stable prior to myosin thick filament formation (S_0_), stable as myosin thick filaments accumulate (S_1_), and contractile (C). Scale bar is 4 μm. **b** Ensemble averaged energy dissipated per unit length, $\overline {\Delta s} T$, as a function of time (blue) and number density of myosin thick filaments counted as a function of time (black). Blue dots and shaded areas are mean ± standard deviation of *N* = 19 filaments tracked in a single experiment. Experiment is broken into three phases, S_0_, S_1_, and C. Red dashed lines indicate slopes measured in each state. **c** Means ± standard deviation for slopes of entropy in states S_0_, S_1_, and C for *N* = 4 experiments (red) and *N* = 3 simulations (blue). The slope reported for each experiment, denoted by a different symbol, is itself an average over *N* = 20, 20, 19, and 20 filaments, respectively, and each simulation is an average over *N* = 100, 152, and 133 filaments, respectively. Each slope is normalized to the slope of S_1_ in that experiment or simulation. For experiments, *p* < 10^−4^ between slopes S_0_ and S_1_, and *p* = 0.011 between slopes S_1_ and C. For simulations, *p* = 0.033 between slopes S_1_ and C. **d** Dissipation energy density as a function of myosin number density in state S_1_ for *N* = 3 experiments, indicated by different symbols. Black line and shaded area are mean ± standard deviation across experiments. **e** Similar to **b**, but showing filament bending energy per unit length, $\overline {\Delta \varepsilon _{\rm bend}}$, in blue. **f** Similar to **c**, but for filament bending energy slopes. For experiments, *p* < 10^−5^ between slopes S_0_ and S_1_, and *p* = 0.001 between slopes S_1_ and C. For simulation, *p* < 10^−4^ between slopes S_1_ and C. **g** Similar to **d** but for filament bending energy. Insets for **b**–**g** show recapitulation of data in main figure by agent-based simulations for *N* = 3 simulations. In the simulations, there is no S_0_ phase because myosin is added immediately at *t* = 0. All *p*-values measured using a two-sided Kolmogorov–Smirnov test

Using experiments where only 2% of filaments are fluorescently labeled, individual filaments are tracked over time (Fig. 4a). Each filament’s shape is decomposed into normal modes at each time point, yielding a time series of mode amplitudes. Filament dynamics are represented by the trajectory of a point in a phase space spanned by the mode amplitudes, ${\mathbf{a}}\left( t \right) = \left( {a_1\left( t \right),a_2\left( t \right), \ldots } \right)$ (Supplementary Figure 8). Such phase space trajectories have recently been used to identify broken detailed balance in mesoscopic biological systems^39–41^ and to calculate rates of energy dissipation in open biochemical systems^42,43^. We use the phase space trajectories to quantify the entropy produced over time using a formulation based on a Langevin equation for the bending modes^44^. Using natural units, the total entropy produced up to a time *t* is given by

1 $$\begin{array}{*{20}{c}} {\Delta S\left( t \right) = \mathop {\int }\limits_0^t {\mathrm{d}}\tau \ {\dot{\mathbf a}}^T\left( \tau \right){\mathbf{D}}^{ - 1}{\mathbf{v}}^{ss}\left[ {{\mathbf{a}}\left( \tau \right)} \right]} \end{array}$$

where ${\mathbf{v}}^{ss}\left[ {{\mathbf{a}}\left( \tau \right)} \right]$ is the steady state phase space velocity estimated using the entire trajectory, and ${\dot{\mathbf a}}\left( \tau \right)$ is the instantaneous phase space velocity. **D** is the diffusion matrix that enters into the Fokker–Planck equation associated with the underlying Langevin equation for the mode amplitudes. For simplicity, we estimate **D** from the drag coefficients of a slender rod^45^. We verify our calculations and approximations by checking that a control system obeys the detailed fluctuation theorem^46^ (Supplementary Note 3, Supplementary Figure 9).

Using the above formalism, we calculate the average total energy dissipated per unit filament length over time as $\overline {\Delta s} \left( t \right)T$, where Δ*s*(*t*) = Δ*S*(*t*)/*L*, *L* is the filament length, *T* is the temperature of the surrounding medium, and the bar denotes an ensemble average taken at each time point. We find that the actomyosin system exhibits three distinct phases of energy dissipation that correspond to the three states of actomyosin discussed above (Fig. 2d). In these experiments, myosin accumulates over time, and the three states coincide with changes in the number of tracked myosin thick filaments over time (Methods). The first state is a passive state distinct from thermal states due to the presence of myosin dimers that have not yet formed myosin thick filaments (S_0_). The second is an active, non-contractile state as myosin thick filaments begin to appear (S_1_). The third is the contractile state (C) where myosin thick filaments begin to aggregate and thus the number of tracked thick filaments decreases (Fig. 4b, black). Here, the peak in myosin number density (*ρ*_c_) is the same as for independent experiments^16^, and thus a steady state assumption is applied. State S_0_ shows a small increase in energy dissipated, followed by a large increase in the rate of energy dissipation during state S_1_. State C shows a decreased energy dissipation rate (Fig. 4b, c). As myosin accumulates in time, we then replot $\overline {\Delta s} T$ against myosin thick filament number density. Within state S_1_, total energy dissipation as a function of myosin number density across experiments collapses along a single curve until the system gets closer to the contractile regime (Fig. 4d). These results are replicated using agent-based simulations (Fig. 4b–d, Supplementary Note 2, Supplementary Table 1, Supplementary Movie 4, 5).

Having investigated the role that activity plays in dissipating mechanical energy, we next sought to understand how activity also stores mechanical energy in the system via filament bending. To this end, we measured the change in bending energy per unit filament length as myosin accumulates, $\overline {\Delta \varepsilon _{\rm bend}} = \overline {\Delta { E}_{\rm bend}/L}$ (Fig. 4e, Methods). As with the dissipation energy, we take the ensemble average across filaments at each time point. We again observe three distinct regimes in time, where the actin bending energy does not change during state S_0_ and increases rapidly in state S_1_ (Fig. 4f). In state C, bending energies remain elevated but decrease nominally. This may be attributed to filament severing in experiment^15,47^, although it is not necessary as simulations without severing show similar results (Fig. 4e, f, insets, Supplementary Figure 10). In simulation, it can be observed that upon the cessation of contractile flow, filaments are polarity sorted with motors at filament barbed ends and bends are released^48^ (Supplementary Movie 6). Again, plotting bending energy as a function of myosin number density in state S_1_ collapses experiments along a single curve as the system approaches contractility (Fig. 4g).

Thus, the non-contractile state (S) dissipates the most energy per unit time as measured through the rate of entropy production. As the stable (S) and contractile states (C) have different entropy production rates, we sought to determine if there was a difference in the underlying actomyosin interactions that produce these rates.

### Transverse actomyosin motions underlie maximal dissipation

Active transverse fluctuations and F-actin bending may suggest that myosin and F-actin are not aligned, in contrast to the canonical model for their interaction of anti-parallel filament sliding. To explore this, we quantify the extent of axial vs perpendicular actin motions and compare them to myosin motions.

**Fig. 5:**
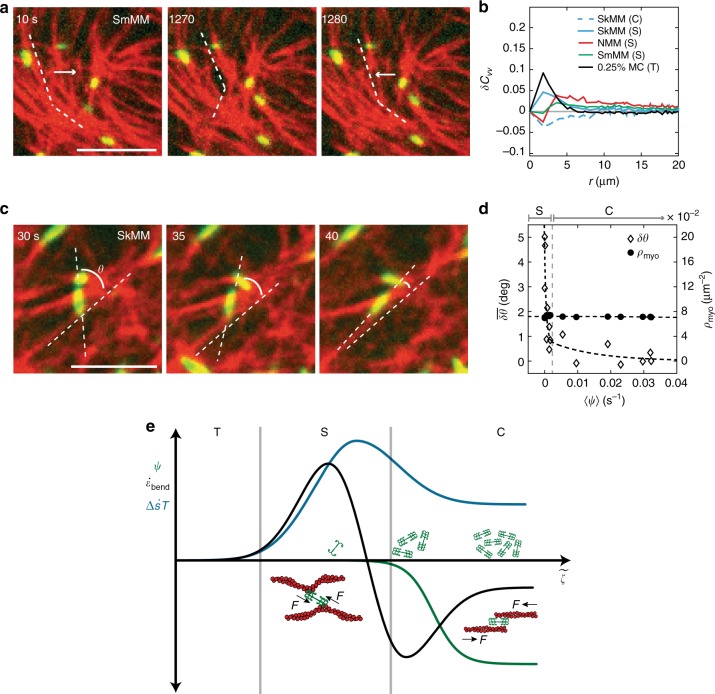
Misaligned myosin bends F-actin perpendicularly in stable actomyosin. **a** Motions of F-actin within an actomyosin network. Images of 40 nM smooth muscle myosin (green) embedded within ~2μM F-actin network (red). White dotted lines indicate alignment of F-actin. White arrows indicate direction of motion of F-actin. Scale bars are 5 μm. **b** Anisotropic velocity-velocity autocorrelation, *δC*_*vv*_. Averages are taken across several experiments (*N*_SkMM_ = *N*_0.25%MC_ = *N*_0.15%MC_ = 3, *N*_SmMM_ = 5, *N*_NMM_ = 2), where each experiment is represented by its own temporal average. The colors represent different experimental conditions. **c** Images of ~2μM actin and 40 nM skeletal muscle myosin. Angle *θ* is measured between the myosin thick filament and the underlying F-actin. Scale bar is 5 μm. **d** Ensemble average change in angle $\overline {\delta \theta (t)} = \overline {\theta (t) - \theta \left( \infty \right)}$ of myosin thick filaments (open diamonds) and mean myosin number density (filled dots) as functions of *ψ*, which increases from 0 to 0.04 s^−1^ as time increases. Dotted lines are guides for the eye. Stable (S) and contractile (C) states indicated above plot. **e** Schematic phase diagram showing how dissipation rate (blue), bending energy rate (black), and *ψ* (green) all change as activity, $\tilde \zeta$, increases. An increase in $\tilde \zeta$ coincides with an increasing myosin density, indicated by the green myosin cartoons. Thermal, stable, and contractile states are indicated by T, S (S_0_ & S_1_), and C, respectively. In the stable state, a schematic representation of myosin’s perpendicular effects on F-actin are shown. As $\tilde \zeta$ increases, myosin is shown sliding anti-parallel filaments

To quantify the extent to which non-contractile networks exhibit perpendicular bending motions, we measure the anisotropic velocity autocorrelation, defined as $\delta C_{vv}\left( r \right) \equiv \langle C_{vv}^ \bot \left( {r,t} \right)/C_{vv}^ \bot \left( {0,t} \right) - C_{vv}^\parallel \left( {r,t} \right)/C_{vv}^\parallel \left( {0,t} \right)\rangle _t$ (Supplementary Methods). Positive values indicate enhanced perpendicular fluctuation autocorrelations; negative values indicate enhanced parallel fluctuation autocorrelations. We find that all stable systems, regardless of myosin isoform, exhibit greater fluctuations perpendicular to the filament axis, in stark contrast with contractile systems that show larger autocorrelations parallel to the filament’s axis as would be expected for sarcomeric contraction^8,49^ (Fig. 5a, b). We name these reversible, myosin-derived transverse fluctuations 'plucking' (Supplementary Movie 7).

Using a light activation assay^31^ with skeletal muscle myosin II (SkMM), we induce contractility at a constant myosin density. 405 nm light inactivates blebbistatin, an ATPase inhibitor, thereby activating myosin in the area of illumination. During contraction, we measure the relative angle between SkMM thick filaments and the actin it decorates (Fig. 5c). The extent of contractility is measured by an increase in *ψ* (Fig. 2). We find that as the magnitude of *ψ* increases, we see a rapid change in the relative angle (*δθ*) between actin and myosin (Fig. 5d). We therefore attribute the enhanced perpendicular fluctuations of actin in a non-contractile state, and therefore the enhanced entropy production rate, to the variation in the relative angles between individual myosin thick filament assemblies and F-actin. Thus, we append our original metric for activity to include both the density of thick filaments *ρ*, but also a wider spectrum of actomyosin interactions which is quantified by the relative angle between motors and filaments, *θ*, i.e. $\zeta \left( \rho \right) \to \tilde \zeta \left( {\theta ,\rho } \right)$ (Fig. 5e).

## Discussion

Through the engineering of an active biomimetic material, we identify a structurally dynamic phase of actomyosin absent of contractile flow or filament turnover at an intermediate level of activity. These dynamics include myosin-driven fluctuations in both the nematic director and F-actin density. Active nematic theory provides a general framework for understanding the coupling between these fluctuations. However, we find that the model does not fit at all length-scales. For low *k*_⊥_, we find dramatically increased density autocorrelations due to myosin activity, indicative of an increase in F-actin mobility. A poor fit to the data challenges the applicability of the active gel model, for two possible reasons. First, the active stress *ζ* assumes that forces are applied along the local nematic director. It was not clear a priori, that this motion that is assumed to drive contractility also occurs in a dynamic steady state^16^. Second, the dissipation characterized by a viscosity *η*, may in fact be activity dependent. Thus, we seek to calculate the effect of activity on dissipation in a model independent way, which makes no assumptions on the specific functional form of the active stress.

Entropy and the rate at which it is produced provide a measure for how far a system is from equilibrium^36,50^ and the extent to which energy is dissipated. As bending energies increase immediately upon sequential addition of individual myosin thick filaments (Fig. 4e), and *ρ*_c_ is the same over time as across an ensemble of experiments^16^, we assume the system to equilibrate quickly and therefore apply a steady state framework. Unexpectedly, we find that the rate of entropy production is non-monotonic with increased activity. As the system is driven from equilibrium, the dissipation rate first increases in the stable state and then attenuates in the contractile state. In addition, the work applied, as indicated by the filament bending energy, increases and then decreases in the contractile state. This is likely due to mechanical relaxation via polarity sorting and filament severing^47^ (Supplementary Figure 10, Supplementary Movie 6). Thus, while the contractile state has the highest entropy, it is the stable state in which the rate of entropy production is maximized (Fig. 5e).

While we find that axial motions of F-actin, consistent with the canonical sliding of F-actin in muscle, are associated with contractility, stable fluctuations are dominated by transverse filament deformations (Supplementary Movie 7). Furthermore, these transverse motions occur regardless of myosin isoform, suggesting the generality of these dynamic modes in the active stable state. These reversible F-actin plucking events arise from transient and diverse interactions between non-aligned myosin and F-actin (Fig. 5). A lack of alignment or overlap between myosin and actin filaments would imply that fewer myosin heads may be involved in the generation of active mechanical stress. Indeed, we find that only a few heads are necessary to induce the observed bending energies (Supplementary Figure 2). Thus, this work challenges the prevailing model of molecular motors as dipoles oriented parallel to F-actin, that always yield to contractility. Likewise, the definition of motor-based activity is now more complex; there is a spectrum of interactions that occur in disordered assemblies of myosin and F-actin at the molecular level. That spectrum in turn, may determine network level entropy production and dissipation that stabilizes actively driven materials.

The relationship between motor activity and the accumulation and dissipation of mechanical energy, which determines material stability is complex. The complexity arises from a diversity of motor-filament interactions, and the impact of those interactions on the dissipation of mechanical energy. Our multi-length scale identification and characterization of active stability presents a comprehensive understanding for the dynamics of active biological materials.

## Methods

### Assay construction

Briefly, a phospholipid bilayer composed of 99.8% Egg Phosphatidyl-Choline (EPC, Avanti Polar Lipids) and 0.2% Oregon Green 1,2-Dihexadecanoyl-sn-Glycero-3-Phosphoethanolamine (OG-DOPC, Molecular Probes) is deposited onto a UV-treated or Piranha-treated coverslip. Stabilized F-actin (2 μM) with 0.3 μM fluorescent actin (Alexa-Fluor 568, Molecular Probes) is crowded to the surface of the bilayer using 0.25% methyl-cellulose (Sigma). Skeletal-muscle myosin (10.5 μΜ stock), smooth-muscle myosin and non-muscle myosin II monomers (2.4 μM stock) are then added to the F-actin network. As the actual concentration within a microscopic field of view can vary, the precise concentration of myosin is calculated by visualizing the labelled myosin in experiment, and calculating the density of myosin thick filaments per unit area (number/μm^2^) from the fluorescence images^31,51,52^.

### Myosin isoforms

Smooth muscle myosin is purified from fresh chicken gizzards and non-muscle myosin is purified from fresh human platelets following an ammonium sulfate precipitation protocol^53^. Skeletal muscle myosin (Cytoskeleton Inc) and other isoforms are stained using an Alexa Fluor-647 maleimide (Molecular Probes)^54^. All isoforms are flash-frozen in liquid nitrogen and stored at −80 °C until use. Both smooth muscle and non-muscle isoforms are phosphorylated with myosin light chain kinase prior to freezing. All myosin isoforms are thawed and spun-down prior to use to remove enzymatically inactive myosin^55^. Skeletal muscle myosin is used to assess alignment between thick and thin filaments due to its length (~2 μm). Smooth muscle myosin is used to assess F-actin deformation due to slower motor kinetics. Skeletal and non-muscle isoforms are also used for light activation as they are sensitive to blebbistatin ATPase inactivation.

Confocal microscopy is used to image the dynamics of fluorescent protein with the assay. Images are recorded on a Zyla 4.2 Megapixel sCMOS camera on a Leica microscope and also recorded on a Coolsnap Hq2 CCD Camera on a Nikon Ti Inverted Microscope.

The density of myosin thick filaments is calculated using Imaris (Bitplane, South Windsor, CT). The peaks of fluorescence intensity of myosin-thick filaments are found in each frame, and the total number of peaks is then divided by the total area of the sample to obtain a myosin thick filament density over time.

### Nematic order parameter calculations

The nematic order parameter, *q*, is calculated using custom Matlab code. First, a director field is created from images of fluorescently labeled F-actin^56^. Briefly, fluorescent images are divided into small, overlapping 3.5 μm by 3.5 μm windows, and the local F-actin orientation (director) is calculated for each window, yielding an F-actin director field over an image. To determine the local F-actin director, each window is Gaussian filtered and transformed into Fourier space using a 2D fast Fourier Transform (FFT). The axis of the least second moment was calculated from the second-order central moments of the transformed window, and the angle of the local F-actin director is defined as orthogonal to this axis. Next, the local degree of alignment is calculated between adjacent windows within 3×3 kernels. The local nematic order is calculated for the central window in each kernel using the modified order parameter equation $q = 2\langle \cos ^2\theta - 1/2\rangle$, where *θ* is the difference in F-actin orientation between the central window and the 8 surrounding windows. This process is repeated for all possible 3×3 kernels over an image, yielding a nematic director field with defined director magnitude and orientation for each window over an image. Perfect alignment between adjacent regions within an F-actin network results in an order parameter equal to one. Conversely, orientation differences of 45° (maximum expected for quasi-2D F-actin network) between adjacent regions of the network result in an order parameter equal to zero.

### Flow calculations

Particle image velocimetry (PIV) is applied in Matlab (mPIV, https://www.mn.uio.no/math/english/people/aca/jks/matpiv/) to fluorescent F-actin images, yielding displacement & velocity vector fields.

### Fluctuation autocorrelations

Using custom Matlab code, the local alignment field, nematic order parameter, and density scalar field are extracted for each confocal image. The equal time autocorrelation function for both the nematic director and the density fields are calculated at every position in the image along an axis transverse to the local alignment field, and the results are binned across all time and space for each experimental condition. See Supplementary Note 1 for more details.

Kymographs are made using ImageJ plugin.

### Agent-based simulations

The experiments are simulated in an open source package, Cytosim^34^. Actin filaments are modeled as polar worm-like chains composed of rigid segments of length 0.1 µm. The simulation volume is quasi-3D with periodic boundary conditions along *x* and *y* axis to limit finite size effect. The boundaries along *z*-axis are closed to mimic the action of methyl cellulose. The thickness of simulation volume was set to twice the diameter of an actin filament. The filaments initially grow for a fixed period of time to reach a predetermined length distribution and volume fraction and then relax to reach an equilibrium steady state. The motors are modeled as Hookean springs that can bind with filaments and move along them toward their barbed end. The details regarding implementation of simulation are described in Supplementary Note 3.

### Calculating entropy from experimental data

In order to calculate the entropy produced by individual filaments, we first track individual filaments using the ImageJ plugin JFilament^57^. Using custom MATLAB scripts, we decompose the filament shapes into a set of orthogonal bending modes. The coefficients for each bending mode are then tracked over time in the configurational phase space spanned by the mode coefficients. We coarse-grain the resulting velocity field in phase space^41^ in order to obtain a steady-state velocity to be used in Eq. (1) to calculate the entropy at each time point for every filament, dividing by the filament length to create an intensive variable. The mean entropy across all filaments present at a given time point is taken, and the cumulative sum of this mean is plotted in Fig. 4e. See Supplementary Note 2 for more details.

### Calculating filament bending energy

Filaments are tracked in time as mentioned above and represented as *M* points along the filament. A circle is fit to rolling sections of *n* ≪ *M* points, and the inverse of the radius of the resulting circle is taken as the local curvature of the filament at that point, *κ*. The bending energy of the entire filament is then given by $E_{\rm bend} = \frac{{EI}}{2}\mathop {\int }\limits_0^L \kappa ^2\left( s \right){\mathrm{d}}s$. *EI* is the flexural rigidity of actin^58^. The ends of the filaments are precluded from this measure because points within *n*/2 points of the end cannot have a set of *n* points centered around them.

### Code availability

Code used to production nematic fluctuation correlations and to compute entropy production is available at the following Github repository: https://github.com/lab-of-living-matter/actin-entropy-paper. Code used to measure the nematic order parameter is available at the following Github repository: https://github.com/OakesLab/FFT_Alignment.

## Electronic supplementary material

Supplementary Information

Supplementary Movie 1

Supplementary Movie 2

Supplementary Movie 3

Supplementary Movie 4

Supplementary Movie 5

Supplementary Movie 6

Supplementary Movie 7

Description of Additional Supplementary Files

## Data Availability

Data supporting the findings of this manuscript are available from the corresponding authors upon reasonable request. A reporting summary for this Article is available as a Supplementary Information file.
