# Supplementary material for: Entropy production rate is maximized in non-contractile actomyosin
Source: Nat Commun. 2018 Nov 23;9:4948. doi: 10.1038/s41467-018-07413-5 (PMC6251913; doi:10.1038/s41467-018-07413-5)
Supplement: Supplementary file 1 — Supplementary Information [file 41467_2018_7413_MOESM1_ESM.docx]

**Entropy Production Rate is Maximized in Non-Contractile Actomyosin**

Seara et al.

Supplementary Information

# Supplementary Methods

### Altering network architecture

We alter the extent of depletion forces and the presence of F-actin binding proteins to modulate the nematic director and density fluctuations. To quantify the thermal fluctuations of the F-actin network alone, we crowd F-actin in the presence of 0.25% methylcellulose (MC), which drives the filaments to organize into nematically ordered domains^1^. The nematic alignment is reduced by decreasing the concentration of MC to 0.15% or by coupling the actin filaments to the membrane via FimA2 in the presence of 0.25% MC (Supplementary Figure 2). In this case, coupling to the membrane prevents spontaneous alignment of filaments. The network architecture can also be altered by adding passive crosslinkers; the addition of the actin crosslinking protein α-actinin (1:30 α-actinin:actin) induces bundling of the filaments. Myosin II isoforms, including sk­eletal muscle myosin II (SkMM), smooth muscle myosin II (SmMM), and non-muscle myosin II (NMM), are then added to drive the system out of equilibrium.

### Rescaled nematic order parameter

The scalar nematic order parameter is calculated using the 2D functional form, $\left\langle q \right\rangle=2 \left\langle\cos^{2} \theta-1/2 \right\rangle$, where *θ* is the angle difference between the alignment of the current interrogation window and each of the surrounding adjacent windows in a 3×3 kernel of non-overlapping windows. For the in vitro assay used in this study, the maximum alignment angle difference expected for adjacent interrogation windows is approximately *θ* = 45°, which results in *q* = 0 (maximum disorder). For perfectly aligned regions of the network, there is no difference in alignment (*θ* = 0°) and *q* = 1, indicating perfect nematic ordering. Thus, the 2D form of the scalar order parameter equation varies from *q* = [0, 1] for changes in F-actin alignment from *θ* = [45°, 0°], respectively.

### Calculating Q-tensor

The two-dimensional tensor nematic order parameter, the *Q*-tensor, is a symmetric, traceless tensor given by

$$\begin{aligned} Q_{ij}=\frac{1}{N}\sum_{\alpha} \left( n_{i}^{\alpha}n_{j}^{\alpha}-\frac{\delta_{ij}}{2} \right) \end{aligned}(1)$$

Where the sum runs over the directors of *N* individual mesogens. Experimentally, we do not have access to the exact mesogen directors, so we approximate them using the custom Matlab code described in the main methods to extract the nematic director vector field. We then sum over every 3×3 kernel of vectors to construct the spatial map of *Q.* The scalar nematic order parameter, *q*, is defined as twice the largest eigenvalue of *Q.* See SFig 3.

### Calculating fluctuation correlations

The confocal images are first cropped to 700×700 pixels and registered using StackReg (ImageJ plug-in). Using custom Matlab code^2^, the alignment field and nematic order parameter are extracted from each confocal image, using overlapping interrogation windows (3.5μm × 3.5μm, ~51.5% overlap), thereby defining a local nematic director at the center of each interrogation window (Supplementary Figure 2). The density scalar field is generated by averaging the confocal image intensity values within overlapping interrogation windows (3.5μm × 3.5μm, ~51.5% overlap) for consistency. The custom fluctuation autocorrelation Matlab code steps through each grid position of the nematic director field and projects a new coordinate axis orthogonal to the local nematic director at the current grid position. This projected perpendicular axis will serve as a new 1D coordinate system, along which network fluctuations will be correlated (Fig. 3c). The spatial mean along the axis is calculated and subtracted from each individual value that lies on the axis, resulting in the fluctuations about the mean. The autocorrelation of the fluctuation data along the locally-defined perpendicular axis is computed in Fourier space for each nematic director in the time series. The fluctuation autocorrelation data are binned across all space and time, yielding a single equal-time autocorrelation for the entire time series experiment. Further, different experiments with the same conditions are averaged to obtain the class average autocorrelation series for each type of experiment. The orientation and density fluctuation autocorrelations are performed identically, except the density fluctuations are scalar whereas the orientation fluctuations are vector valued. The analytical forms for these autocorrelations are given in Supplementary Equations 12 and 13 below.

First, we measure the fluctuation in a thermal network (T) composed of short filaments. In this case, the F-actin director fluctuations exhibit *S_nn_*(*k*_⊥_) ~ *k*_⊥_^-2^ scaling at large wave numbers indicating a strong propensity to align. In addition, the density fluctuations display *S__*(*k*_⊥_) ~ *k*_⊥_^-4^ scaling at short lengths, indicating the diffusion and translation of rigid filaments. These results are consistent with active gel theory, which describes the coupling between density and orientation (Supplementary Note 1)^3,4,5^. However, for filaments of lengths comparable to the F-actin persistence length, we find significant deviations from this model. The director fluctuations are attenuated from the *k*_⊥_^-2^ scaling and *S__* at high *k* decays more quickly than the predicted *k*_⊥_^-4^ scaling, both suggestive of strong disorder or dissipative effects. We reproduce these results using the simulation package Cytosim^6^ which indicates the *S__* fluctuation scaling exponent is independent of filament bending stiffness, but depends upon a length-dependent viscous drag (Supplementary Figure 7).

### Anisotropic velocity autocorrelation function

The velocity autocorrelation functions in Fig. 4b in the main text are calculated as follows. For each frame in a movie of an F-actin network, the velocity and alignment fields are calculated as described in the main methods. Each vector is then decomposed into components parallel and perpendicular to the alignment found at its grid point, defined as $v^{\parallel}\left( \mathbf{x}, t \right)= \left| \mathbf{n}\cdot\mathbf{v} \right|,$and $v^{\perp}\left( \mathbf{x}, t \right)=\left| \mathbf{v} \right|- v^{\parallel}$, respectively. The spatial mean is then subtracted to remove short and long wavelength correlations, giving us $\delta v^{\perp}\left( \mathbf{x}, t \right)= v^{\perp}\left( \mathbf{x}, t \right)-\left\langle v^{\perp}\left( \mathbf{x}, t \right) \right\rangle_{V}$, and similarly for $v^{\parallel}$. The velocity autocorrelation function is then given by

$$\begin{aligned} C_{vv}^{\perp}\left( r, t \right)=\frac{1}{A}\int d\mathbf{x}d\mathbf{x}^{\mathbf{'}}\left[ \delta v^{\perp}\left( \mathbf{x}, t \right) \delta v^{\perp}\left( \mathbf{x}^{\mathbf{'}}, t \right) \delta\left( r-\left| \mathbf{x}-\mathbf{x}^{'} \right| \right) \right] \end{aligned}(2)$$

where *A* is the total area of the field of view, *δ*(·) is the Dirac delta function. A similar expression is used for $C_{vv}^{\parallel}$. The anisotropic autocorrelation function is defined as the temporal average of the difference between the normalized perpendicular and parallel autocorrelations, or

$$\begin{aligned} \delta C_{vv}\left( r \right)=\left\langle\frac{C_{vv}^{\perp}\left( r,t \right)}{C_{vv}^{\perp}\left( 0,t \right)}- \frac{C_{vv}^{\parallel}\left( r,t \right)}{C_{vv}^{\parallel}\left( 0,t \right)} \right\rangle_{t} \end{aligned}(3)$$

### Measuring relative angles between myosin thick filaments and F-actin

The angular direction of the myosin was determined using the image analysis toolbox of Matlab. Specifically, we used the regionprops function to find the center of mass of the myosin images. The position of the center of mass was used to determine which myosin falls inside the light shed region and which myosin falls outside the region. The same function also finds the angle in degree between the *x*-axis and the major axis of the ellipse that has the same second-moments as the myosin image. For the direction of actin filaments, we implemented the custom Matlab routine described in the Methods section of the main text to produce a nematic director field estimating the local alignment of F-actin filaments in space. To determine the relative angle between a myosin thick filament and the actin filaments, we choose the nearest F-actin alignment vector from the center of mass of the myosin image. Then, the angle between that vector and the major axis of the corresponding ellipse of the myosin image is calculated.

# Supplementary Note 1 Active gel theory

We consider an active gel model of the actomyosin gel. The dynamics of the system are specified by the local nematic alignment, **n**(**x**, t), density of actin $\rho_{a}$(**x**, t), and the velocity field, **v**(**x**, t), of actin motion. Equation for the dynamics of the nematic director, **n**, comes from standard liquid crystal physics

$$\begin{aligned} \partial_{t}\mathbf{n}= -\mathbf{v}\cdot\nabla\mathbf{n}+\boldsymbol{\Omega n}+\lambda\mathbf{An}+K\nabla^{2}\mathbf{n}+\boldsymbol{\xi,} \end{aligned}(4)$$

where **v** is the flow field of the actin, $\Omega_{ij}=\frac{1}{2}\left( \partial_{i}v_{j}-\partial_{j}v_{i} \right)$ is the vorticity tensor, *λ* is the flow alignment parameter,$A_{ij}=\frac{1}{2}\left( \partial_{i}v_{j}+\partial_{j}v_{i} \right)$ is the symmetrized strain rate tensor. *K* is the Franck elastic constant penalizing local distortions in the alignment vector, and **** (orientation noise) is a random white Gaussian noise with zero mean. The equation of motion for the density field of actin is given by,

$$\begin{aligned} \partial_{t}\rho_{a}= -\nabla\cdot\left[ \rho_{a}\left( \mathbf{v}+v_{0}\mathbf{n} \right) \right]+ D\nabla^{2}\rho_{a}, \end{aligned}(5)$$

where *D* is the diffusion constant, and *v*_0_ is the effective propulsion velocity or mobility of actin due to myosin driven forces. Supplementary Equations 4 and 5 are coupled to the local motion of actin filaments, which is determined from the force balance equation

$$\begin{aligned} \Gamma v_{i}=\partial_{j}\left( \sigma_{ij}^{el}+\sigma_{ij}^{d}+\sigma_{ij}^{a} \right), \end{aligned}(6)$$

where *Γ* is the friction with the substrate, $\sigma_{ij}^{el}$ is the elastic component of stress tensor, $\sigma_{ij}^{d}$ is the

dissipated stress and $\sigma_{ij}^{a}$ is the active stress. The different contributions to the stress tensor are given by:

$$\sigma_{ij}^{el}=-\frac{\lambda}{2}\left( n_{i}h_{j}+n_{j}h_{i} \right)+\frac{1}{2}\left( n_{i}h_{j}-n_{j}h_{i} \right),$$

$$\sigma_{ij}^{d}=\eta A_{ij},$$

$$\sigma_{ij}^{a}=\zeta(\rho)\rho_{a}\left( n_{i}n_{j}-\frac{1}{2}\delta_{ij} \right),$$

where $h_{i}=K\nabla^{2}n_{i}$, *η* is the viscosity parameter, and $\zeta\left( \rho\right)>0$ is the magnitude of the active contractile stress, which is function of the density of molecular motors.

#### Analytical director and density autocorrelation functions

To calculate spatial autocorrelation functions we consider small fluctuations in nematic director about a locally aligned state: $\mathbf{n}=\mathbf{n}_{0}+\delta\mathbf{n}_{\perp}$. We also consider density fluctuations around a constant steady state: $\rho= \rho_{0}+\delta\rho$, and velocity fluctuations, δ**v**, around a stationary state. We derive equations of motion for $\delta\mathbf{n}_{\perp}, \delta\mathbf{v}$, and $\delta\rho$by linearizing Supplementary Equations 4–6 around the steady state (**n**_0_, *ρ*_0_, 0), by choosing $\mathbf{n}_{0}= \hat{\mathbf{x}}$. Transforming to Fourier space, we derive the following equal time autocorrelation function for the director fluctuations (derivation not shown):

$$\begin{aligned} S_{nn}\left( k \right)=\frac{1}{2} \frac{\Delta^{2}}{Kk^{2}+\frac{\zeta k^{2}}{\Gamma+\eta k^{2}}} , \end{aligned}(7)$$

where *k* is the magnitude of the Fourier wave vector, and$\Delta=\mathbf{n}_{\perp}\cdot\boldsymbol{\xi}$. Similarly, the equal time spatial autocorrelation actin density fluctuation is given by:

$$\begin{aligned} S_{\rho\rho}\left( k \right)=\frac{1}{2}\frac{\Delta^{2}k^{2}\left( \rho_{0}v_{0} \right)^{2}}{\left( Dk^{2} \right)^{2}+\left( Kk^{2}+\frac{\zeta k^{2}}{\Gamma+\eta k^{2}} \right)^{2}}\left( \frac{1}{Dk^{2}}+\frac{1}{Kk^{2}+\frac{\zeta k^{2}}{\Gamma+\eta k^{2}}} \right). \end{aligned}(8)$$

For $\rho<\rho_{c}$, where $\rho_{c}$ is the critical myosin density, the system is non-contractile. Therefore the contractile activity is zero, i.e. $\zeta\left( \rho<\rho_{c} \right)=0$. In this limit, $S_{nn}(k)\approx$($\Delta^{2}/2K)k^{-2}$, which is inconsistent with the experimental results in Fig. 3 for low $k$. Similarly, in the non-contractile limit, $S_{\rho\rho}(k)\propto k^{-4}$, inconsistent with experimental data.

# Supplementary Note 2 Agent-based simulations

We simulated the actin networks using the open source package Cytosim^6^ to better understand the microscopic interactions between myosin motors and actin filaments. Individual actin filaments are modeled as worm-like chains (WLC), with a diameter of 0.05 µm and persistence length of 16 µm. Each filament is composed of 0.1 µm long linear rigid segments. We first assemble filaments in order to closely mimic experimental conditions. The boundaries along *x* and *y* are periodic to limit finite size effects, whereas the thickness along *z*-direction is chosen to be 0.1 μm to mimic crowding by methylcellulose. The filaments are initialized as 0.1 µm long seeds that grow at a fixed rate to a predetermined size. The growth rate is chosen from a Gaussian distribution to capture polydisperity in filament lengths. By growing the filaments for a fixed amount of time, we control the final length and number of seeds to fix the volume fraction at 0.5. Once filaments reach their predetermined lengths, we set the growth rate to zero and let the system relax thermally before performing subsequent analysis.

To guide our choice of parameters for the simulation, we quantified the effect of variations in the length of filaments, persistence length, viscosity, and volume fraction on the exponent of *S__* (Supplementary Figure 7). As the isotropic-to-nematic transition for rod like particles occur at extremely low volume fraction^7,8^, the behavior of *S_nn_* is independent of any variation in quantities mentioned above and decays as *k*_⊥_^-2^ for large *k*_⊥_. *S__* is sensitive to some of these parameters. As it is difficult to exactly determine and simulate the physical conditions of the experimental setup, we chose our simulation parameters such that, for an assembly of short filaments, *S__* decays as *k*_⊥_^-4^ for large *k*_⊥_. See Supplementary Table 1 for specific parameters.

# Supplementary Note 3 Entropy Computation

Calculating entropy from experimental conditions requires the tracking individual filaments, decomposing the filament shapes into bending modes, and finally constructing the resulting velocity field in configurational phase space. These steps are elaborated on below.

#### Filament tracking and mode decomposition

We track filaments over time in a sparsely labeled in vitro2D actin network using the ImageJ plugin JFilament^9^. The shape of a thin rod of length *L* at time *t* can be described by its vertical displacement off a horizontal, *u*(*s,t*), where $s\in[-L/2, L/2]$ is the contour-length. The dynamics of *u*(*s,t*) can be solved as an eigenvalue problem^10^, $\partial_{ssss}\phi_{n}=\lambda_{n}^{4}\phi_{n}$, where $\lambda_{n}=k_{n}/L$ and $k_{n}\approx(n+1/2)\pi$, and

$$\begin{aligned} \phi_{n}\left( s \right)=\left\{ \begin{matrix} L^{-\frac{1}{2}} \left( \frac{\cos\left( \frac{k_{n}s}{L} \right)}{\cos\left( \frac{k_{n}}{2} \right)}+\frac{\cosh\left( \frac{k_{n}s}{L} \right)}{\cosh\left( \frac{k_{n}}{2} \right)} \right) n=1,3,5,\ldots\\ L^{-\frac{1}{2}}\left( \frac{\sin\left( \frac{k_{n}s}{L} \right)}{\sin\left( \frac{k_{n}}{2} \right)}+\frac{\sinh\left( \frac{k_{n}s}{L} \right)}{\sinh\left( \frac{k_{n}}{2} \right)} \right) n=2,4,6,\ldots\end{matrix} \right\}. \end{aligned}(9)$$

The transverse displacements of the filament*, u*(*s,t*), can now be written as an expansion in these modes, $u\left( s,t \right)= L^{1/2}\sum_{n=1}^{\infty} a_{n}\left( t \right)\phi_{n}(s)$. Applying inverse Fourier transform, the mode amplitudes can be solved as,

$$\begin{aligned} a_{q}\left( t \right)= L^{-\frac{1}{2}}\int_{-\frac{L}{2}}^{\frac{L}{2}} ds^{'} \phi_{q}\left( s^{'} \right) u\left( s^{'}, t \right)= -L^{-\frac{1}{2}}\int_{-\frac{L}{2}}^{\frac{L}{2}} ds^{'}f_{q}\left( s^{'} \right) \theta\left( s^{'},t \right) \end{aligned}(10)$$

where integration by parts was used in the second equality, $\theta\left( s,t \right)= \partial_{s}u\left( s,t \right)$ is the tangent angle along the arc-length over time, and $f_{q}\left( s \right)\equiv\int\phi_{q}\left( s \right) ds$ is

$$\begin{aligned} f_{q}\left( s \right)=\left\{ \begin{matrix} \frac{\sqrt{L}}{k_{n}} \left( \frac{\sin\left( \frac{k_{n}s}{L} \right)}{\cos\left( \frac{k_{n}}{2} \right)}+\frac{\sinh\left( \frac{k_{n}s}{L} \right)}{\cosh\left( \frac{k_{n}}{2} \right)} \right) n=1,3,5,\ldots\\ \frac{\sqrt{L}}{k_{n}}\left( \frac{\cos\left( \frac{k_{n}s}{L} \right)}{\sin\left( \frac{k_{n}}{2} \right)}+\frac{\cosh\left( \frac{k_{n}s}{L} \right)}{\sinh\left( \frac{k_{n}}{2} \right)} \right) n=2,4,6,\ldots\end{matrix} \right\} \end{aligned}(11)$$

The final expression in Supplementary Equation 10 is used to calculate the mode amplitudes, *a_q_*(*t*). The units of the normal mode expansion have been chosen to give *a_q_*(*t*) units of length.

#### Theoretical description of system

The coupled system of normal modes evolves according to the following overdamped Langevin equation

$$\begin{aligned} \dot{\mathbf{a}}=\mathbf{Aa}+\boldsymbol{F\xi} \end{aligned}(12)$$

where $\mathbf{a}\left( t \right)=(a_{1}\left( t \right), a_{2}(t),\ldots)$ is the phase space vector describing the shape of the filament at time *t* and $\boldsymbol{\xi}$ is Gaussian distributed white noise. The applied forcing $\mathbf{Aa}$ contains both conservative and non-conservative contributions, $\mathbf{Aa}= -\nabla V\left( \mathbf{a} \right)+\boldsymbol{\zeta}$, where $\nabla_{i}=\partial/\partial a_{i}$. The non-conservative contribution, $\boldsymbol{\zeta}$, contains the active forces acting on the filament due to molecular motors.

The probability distribution of the system in phase space, $p(\boldsymbol{a}, t)$, evolves under the corresponding Fokker-Planck equation:

$$\frac{\partial p\left( \mathbf{a}, t \right)}{\partial t}= -\boldsymbol{\nabla}\cdot\mathbf{j}\left( \mathbf{a}, t \right)$$

$$= -\boldsymbol{\nabla}\cdot(\mathbf{Aa} p(\mathbf{a},t)-\mathbf{D}\nabla p\left( \mathbf{a}, t \right),$$

where 2**D** = **F**^T^**F**, defines the diffusion matrix. The phase space velocity, $\dot{\boldsymbol{a}}$, can be written in terms of the phase space current $\mathbf{j}\left( \mathbf{a}, t \right)$^11^

$$\begin{aligned} \dot{\mathbf{a}}= \frac{\mathbf{j}\left( \mathbf{a}, t \right)}{p\left( \mathbf{a},t \right)}=\mathbf{Aa}-\boldsymbol{D\nabla}\ln\left( p\left( \mathbf{a}, t \right) \right) . \end{aligned}(13)$$

Entropy of each specific stochastic trajectory of the filament is given as^12^ $S_{sys}\left( t \right)\equiv-\ln\left( p\left( \mathbf{a}, t \right), t \right)$. Taking a time derivative and assuming we are in steady state ($\partial_{t}p^{ss}=0$), we find

$$\frac{dS_{sys}\left( t \right)}{dt}= -\frac{1}{p}\left( \frac{\partial p}{\partial t}+\boldsymbol{\nabla}p\cdot\frac{\partial\mathbf{a}}{\partial t} \right)$$

$$= -\left( \boldsymbol{\nabla}\ln p \right)\cdot\frac{\partial\mathbf{a}}{\partial t}$$

$$={\dot{\mathbf{a}}}^{T}\mathbf{D}^{-1}\mathbf{v}^{ss}- {\dot{\mathbf{a}}}^{T}\mathbf{D}^{-1}\left( \mathbf{Aa} \right)^{ss},$$

using Supplementary Equation 13 with $p^{ss}$ to define the steady-state phase space velocity $\mathbf{v}^{ss}$. While this constitutes the rate of change of the entropy for the system, there is also the entropy associated with the medium. Considering the first law of thermodynamics, *dU = dW + dQ*. At steady-state, *dU*=0, so $\dot{Q}= -\dot{W}=\partial_{t}(F\mathbf{a})$. In our case, $F=\mathbf{Aa}$ and has no explicit time dependence. Therefore,

$$\frac{dS_{med}\left( t \right)}{dt}=\frac{\dot{Q}}{T}=\frac{1}{T}\left( \frac{\partial\mathbf{a}}{dt} \right)^{T}\mathbf{Aa}.$$

Considering Stokes-Einstein, we recognize the rate of change of entropy for the medium is exactly equal to the last term in the rate of change of entropy for the system. This is the entropy flux, and the first term is the entropy production rate. Taking a time integral after adding the two contributions, we arrive at Equation 1 in the main text for the total change of entropy in both the system (filament) and surrounding medium,

$$\begin{aligned} \Delta S(t)=\int_{0}^{t} d\tau{\dot{\mathbf{a}}}^{T}\left( \tau\right)\mathbf{D}^{-1}\mathbf{v}^{ss}\left[ \mathbf{a}\left( \tau\right) \right]. \end{aligned}(14)$$

#### Calculating ΔS from experimental data

Beginning from a time series of mode coefficients obtained as described above, we estimate the velocity using the Stratonovich convention for stochastic differential equations^12^

$$\dot{\mathbf{a}}(t)= \frac{\mathbf{a}\left( t+\delta t \right)-\mathbf{a}(t-\delta t)}{2 \delta t}$$

The steady state velocity is found by first coarse-graining the *N*-dimensional phase space^13^ into bins of size Δ*a*. Each position along the trajectory is then assigned to a bin, *α*, and the velocity at that point is computed. The steady state velocity is then simply the average velocity in each bin. Using Latin superscripts to denote time indices and Greek subscripts to denote vector components, we write

$$v_{\mu}^{ss}\left( \alpha\right)=\left\{ E\left( \frac{a_{\mu}^{i+1}- a_{\mu}^{i-1}}{2\delta t} \right)| \mathbf{a}^{i}\in\alpha\right\}.$$

The instantaneous velocity is then simply the velocities that are used to calculate $\mathbf{v}^{ss}$. The diffusion matrix is estimated as a constant that depends on the length of the filament. Using the Stokes-Einstein relation, $D={k_{B}T}/\gamma$, where *γ* is the transverse drag coefficient for a thin rigid rod of length *L* with radius *r*, given by^14^

$$\gamma=\frac{4\pi\eta L}{\ln\left( \frac{L}{2r} \right)+0.84}.$$

#### Testing the Detailed Fluctuation Theorem

The Detailed Fluctuation Theorem (DFT) states that the relative probability of observing a forward trajectory that creates a fixed amount of entropy compared to a reversed trajectory destroying that amount of entropy satisfies the following expression^15^

$$\begin{aligned} \frac{P\left( +\Delta S \right)}{P\left( -\Delta S \right)}=e^{{\Delta S}/{k_{B}}}. \end{aligned}(15)$$

To verify that the entropy we calculate is correct, we check that it obeys the DFT for a system with sufficient statistics. Specifically, we use high resolution data of a beating axoneme^16, 17^ analyzed using the same methods described above to analyze F-actin filaments. We indeed find that plotting the natural logarithm of Supplementary Equation 15 gives a slope of 1, as done previously for driven colloidal systems^18^ (Supplementary Figure 9).

# Supplementary Table 1 Agent-based Simulation Parameters

| **Parameter** | **Value** |
| --- | --- |
| Mean Filament Length | 2 µm – 10 µm |
| Filament diameter | 0.05 µm |
| Filament segmentation | 0.1 µm |
| Persistence Length | 16 µm |
| Volume fraction | 0.5 |
| Motor speed | 1 µm/sec |
| Stall Force | 0.5 pN |
| Motor stiffness | 1 pN/µm |
| Motor dimension | 0.5 µm |
| Simulation dimension | 20 µm × 20 µm × 0.1 µm |
| Environment viscosity | 1 N s/m^2^ |
| Time step | 10^-3^ s |
| Total simulation time | 30 s – 90 s |

# Supplementary Figures


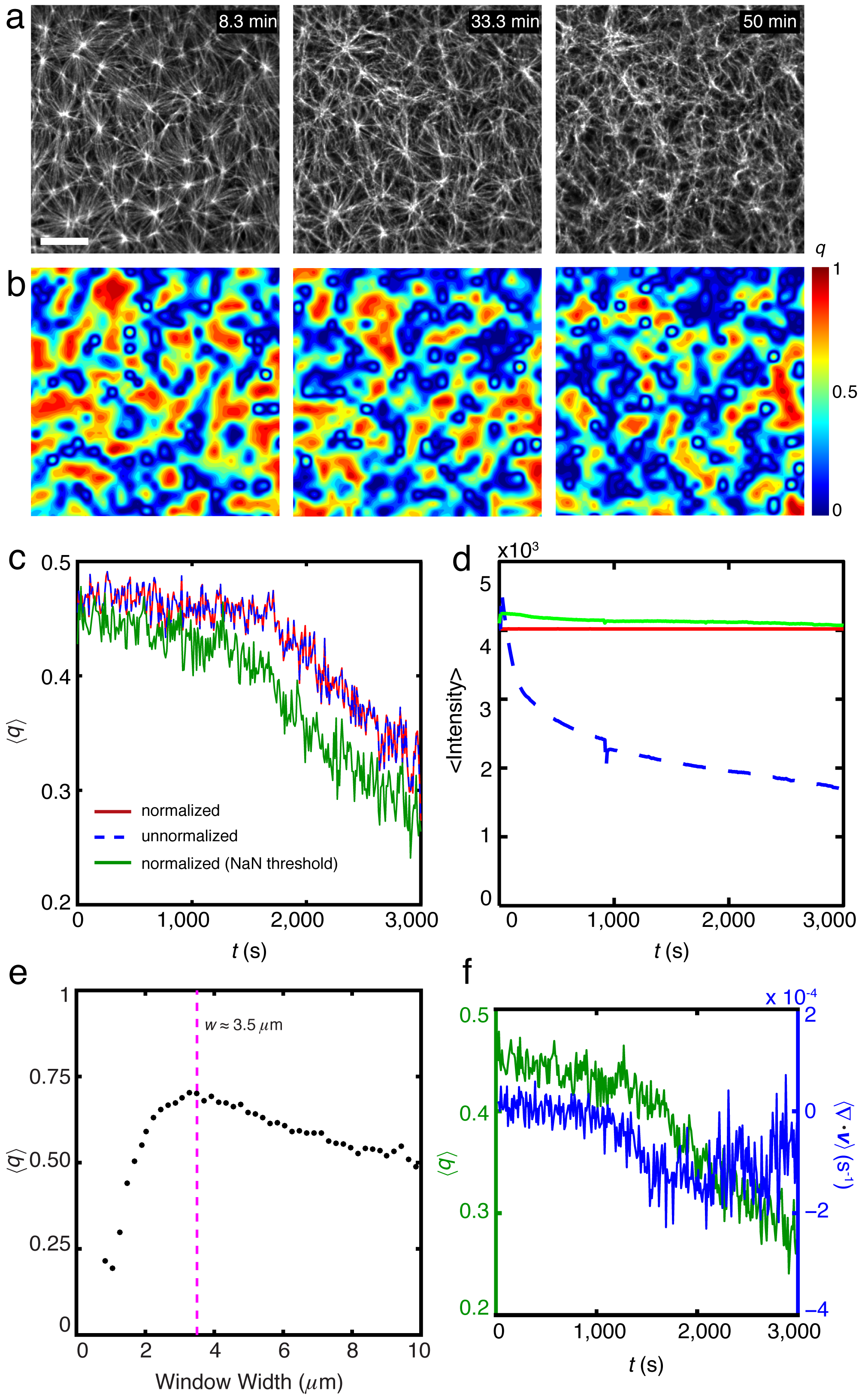


**Supplementary Figure 1 Effect of image analysis on average nematic order parameter over time**

(a) Montage of evolution of stable F-actin network over time. Scale bar is 10 μm (b) Average nematic order parameter for corresponding images in (a). (c) Change in average nematic order parameter over time for the network shown in part (a). Compares the effect of different intensity normalization schemes on the image. We tested the effects of normalized intensity (red solid line), unnormalized intensity (blue dashed line), and normalized intensity, where intensity values were thresholded such that values below a percentage of the maximum were replaced by NaN’s (green solid line). (d) Plots of the intensity of the movie over time using the same normalization schemes as in (c). (e) Effect of window size on the mean nematic order parameter. The local nematic order parameter is calculated over the nematic director field and averaged over all space, yielding a single value that captures the mean nematic order of the F-actin network s (Methods). Here, we vary the size of the interrogation window and use the size corresponding to the maximum average nematic order parameter found throughout the rest of the work, corresponding to windows of size 3.5 µm. (f) Change in average order parameter and average velocity divergence over the course of the movie shown in (a), similar to Fig 2b.


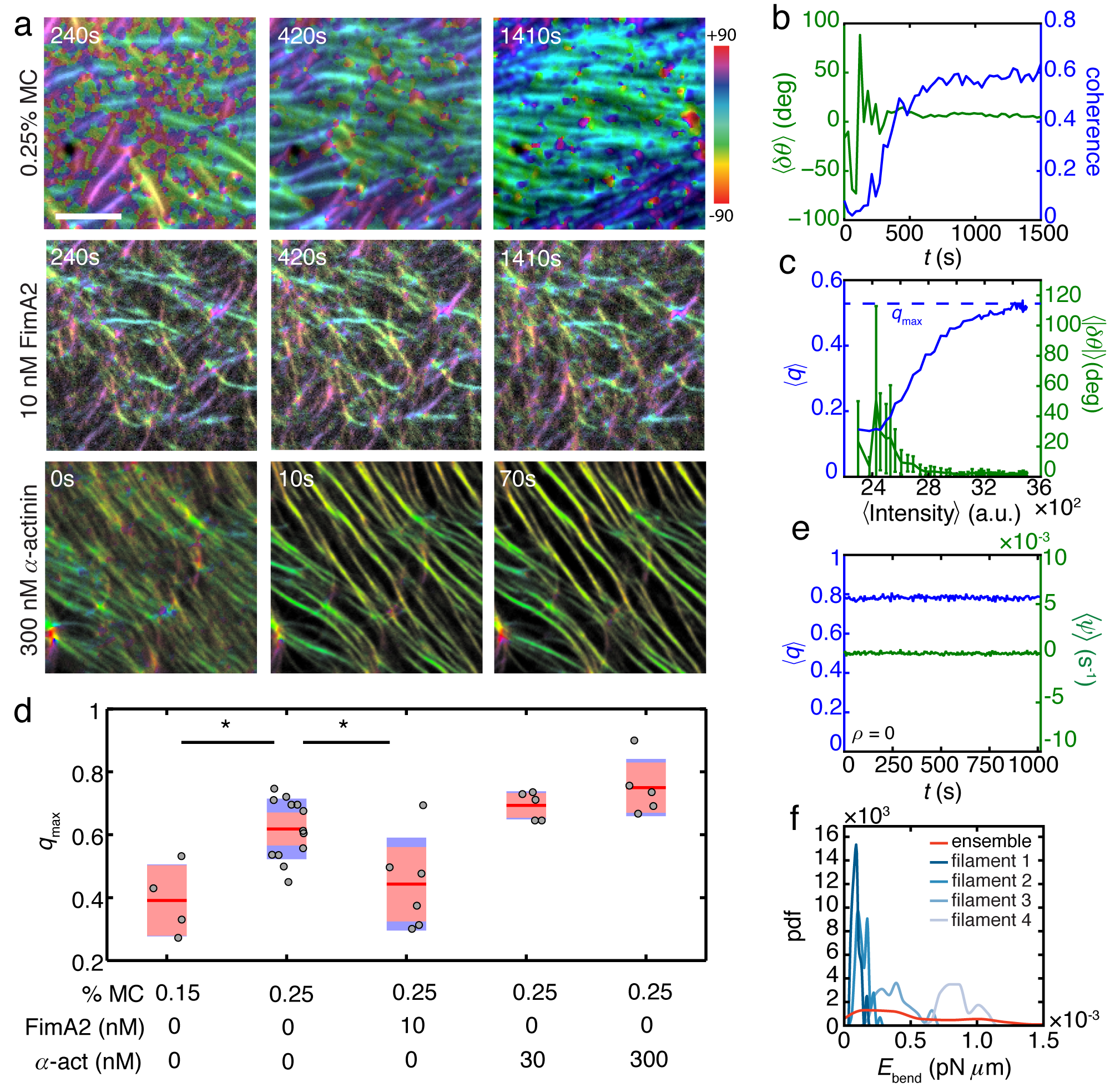


**Supplementary Figure 2 Assembly of in vitroF-actin network**

(a) Montage of F-actin assembly under various conditions: 0.25% methylcellulose (top), 10 nM fimbrinA2 (middle), and 300 nM α-actinin (bottom). Filaments are colored according to their orientation using the ImageJ plugin OrientationJ. Scale bar is 5 μm. (b) For 0.25% MC, the average fluctuation in angle around the mean orientation varies widely at short times, but reaches a steady state as the coherency of the sample increases. The coherency defines how aligned a region of interest is — 1 indicates a perfectly aligned sample, and 0 indicates an isotropic sample (see <http://bigwww.epfl.ch/demo/orientation/)>. (c) Average nematic order parameter, *q*, and average change in angle of the actin filaments as their fluorescent intensity increases during initial crowding. (d) Maximum nematic order parameter for 5 different experimental conditions. Red line indicates mean, red region denotes inner 50% of data and blue region is inner 90% of data. (e) Time evolution of both the scalar order parameter (blue) and velocity field divergence (green) for the thermal system after crowding is completed. Both measures show no temporal changes over 1000 s. (f) Probability density function of F-actin bending energy for an ensemble of filaments at a single time point (N=50, red) and four individual filaments tracked over time (*t* = 120-210s, blue). Individual filaments do not sample the entire range of bending energies available to an ensemble of filaments.


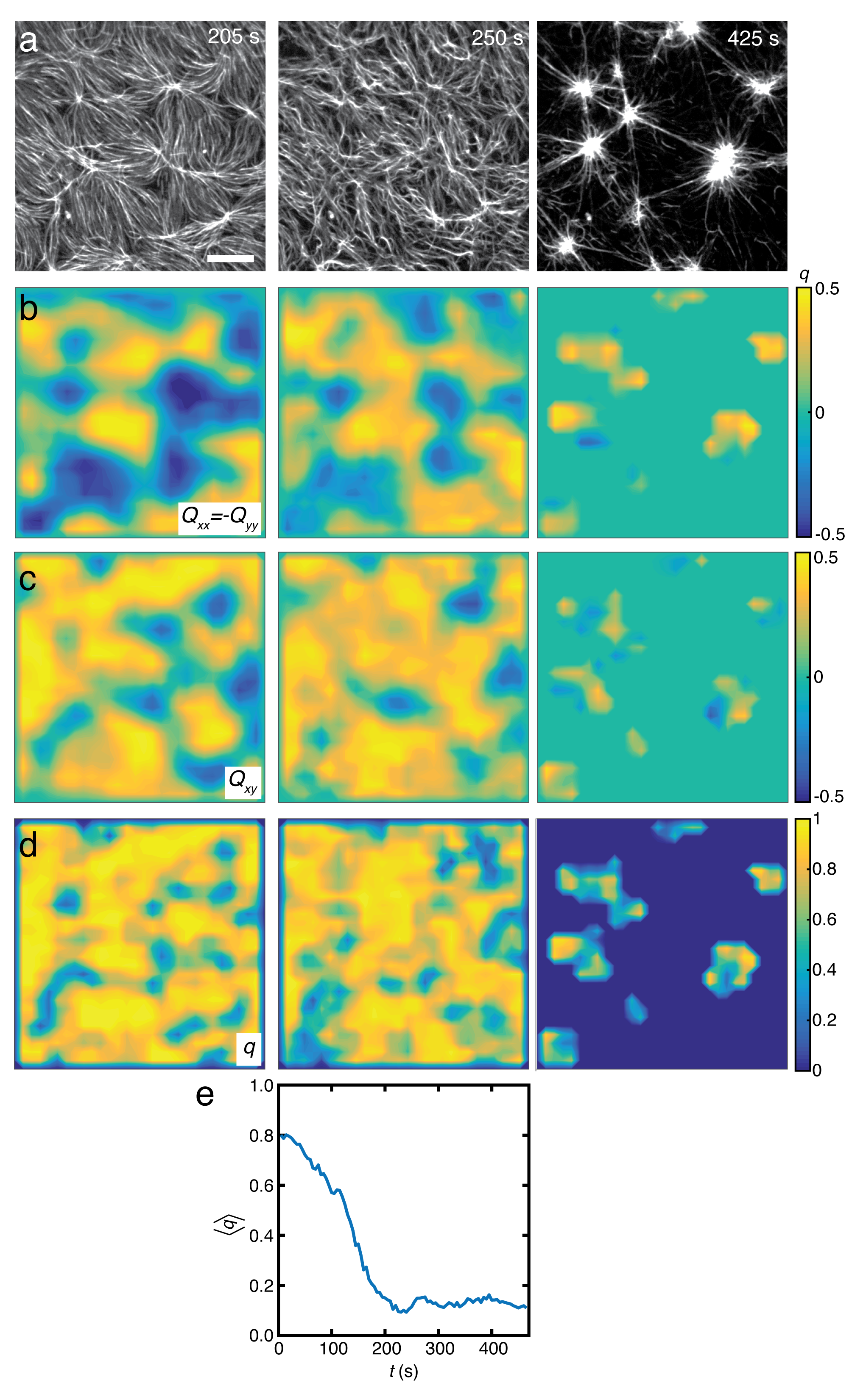


**Supplementary Figure 3 Nematic tensor order parameter**

(a) Time evolution of contractile network depicted in Fig 2. Scale bar is 10 μm. (b) Diagonal elements of 2D **Q**-tensor calculated as described above. Only *Q_xx_* shown because **Q** is traceless, indicating that *Q_yy_*=-*Q_xx_* (c) Off-diagonal elements of 2D Q-tensor. Only *Q_xy_* is shown because **Q** is symmetric, indicating that *Q_xy_* = *Q_yx_*. (d) Scalar order parameter, *q*, measured as twice the largest eigenvalue of **Q***.* (e) Time evolution of scalar order parameter as measured by the eigenvalue of the **Q** tensor shows a similar evolution as the scalar order parameter as measured using the formula used in the main text, $\left\langle q \right\rangle=2 \langle\cos^{2} \theta-1/2\rangle$.

**
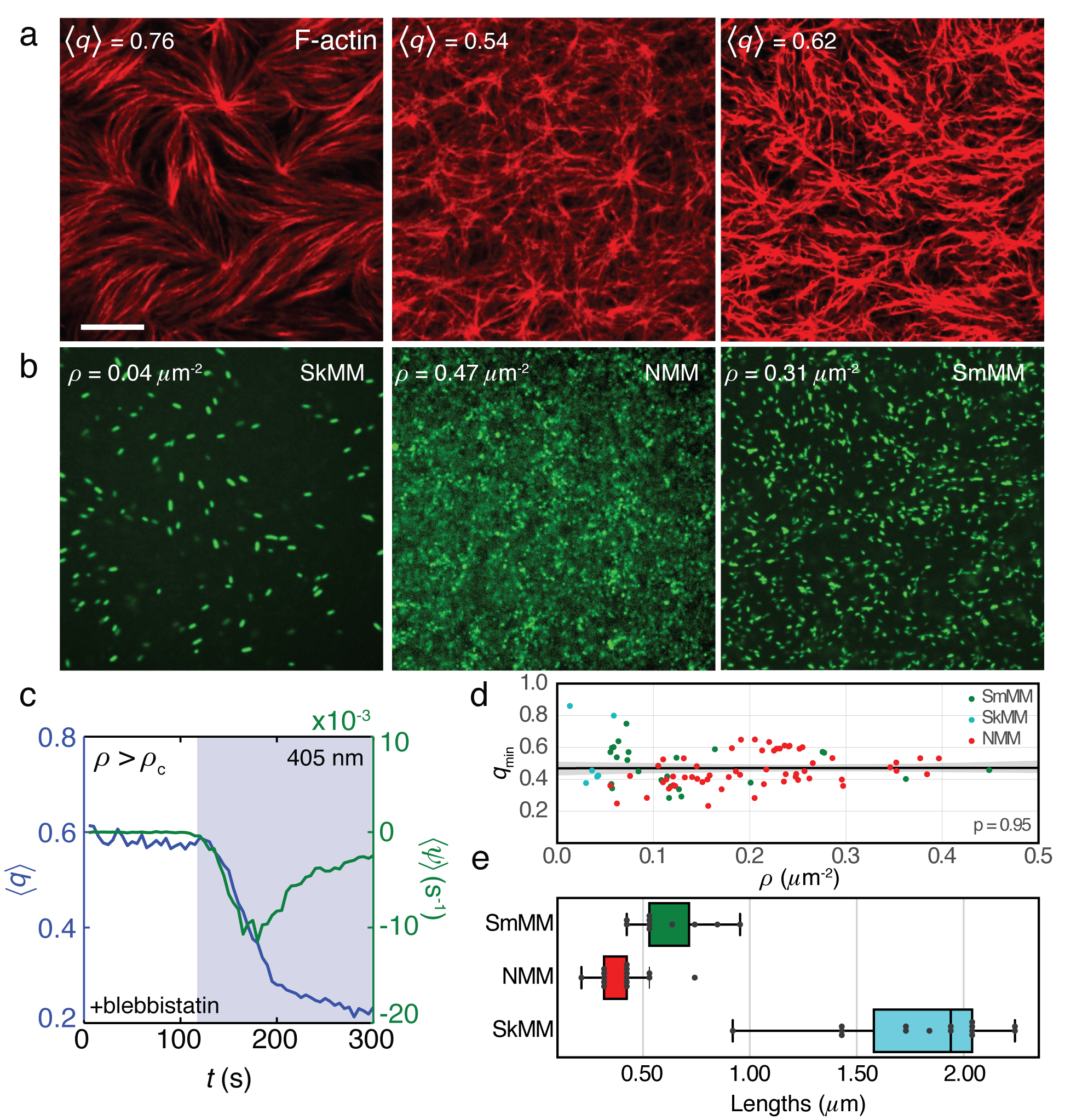
**

**Supplementary Figure 4 Reduction in average nematic order parameter in a non-contractile F-actin network**

(a) Fluorescent images of F-actin in networks with different myosin concentrations and isoforms show a qualitative change in actin architecture with active stresses. * *= 0.04 μm^-2^ of SkMM (left), * *= 0.39 μm^-2^ of NMM (middle) and *ρ* = 0.31 μm^-2^ of SmMM (right). Scale bar is 10 μm and is the same across all images. (b) Fluorescent images of myosin corresponding to images in (a). (c) Spatially averaged nematic order (blue) and divergence of the velocity (green) for a contractile F-actin network * * *ρ*_c_) where myosin molecular motor dimers are already added and are blebbistatin-inactivated at *t*=0. (d) Minimum nematic order parameter achieved for different concentrations of the different myosin isoforms used above for systems with an average velocity divergence of zero. Black line and shaded area indicates linear regression of all data points ± 95% confidence bounds. p=0.95 for the null hypothesis that the line has slope=0. (e) Box plot indicating the length distribution of all myosin thick filament isoforms used, showing a much longer mean thick filament length for SkMM than for NMM or SmMM. The size of NMM approaches the diffraction limit of light used to visualize the thick filaments, making length quantification difficult. Box shows inter-quartile of data, whiskers show extent of the rest of the data, and black line inside of box shows median.


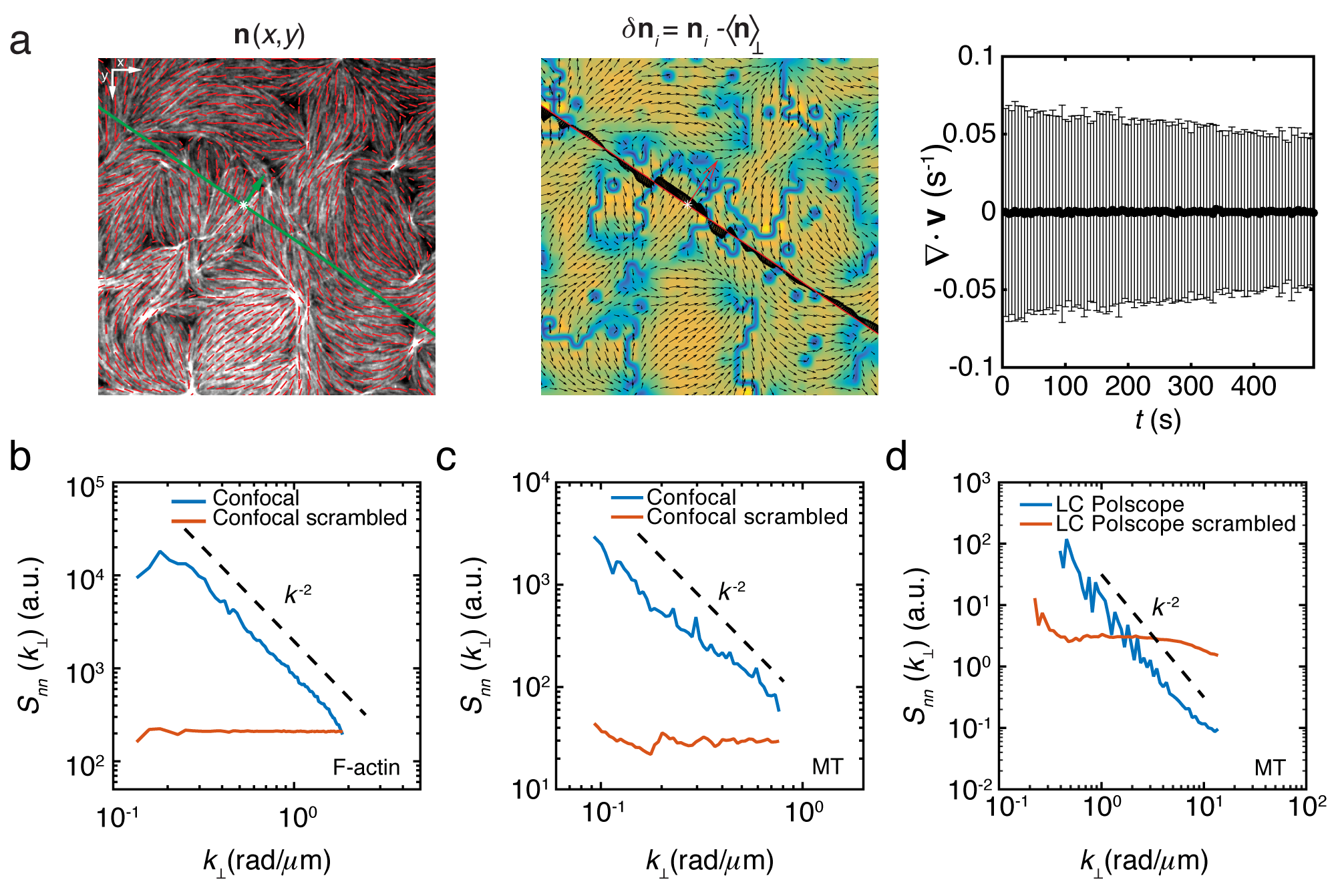


**Supplementary Figure 5 F-actin fluctuation autocorrelation method and tests**

(a) Fluctuation autocorrelations are calculated at every grid position with a defined alignment vector, and a local coordinate system is defined perpendicular to the alignment vector at that position (left). The nematic director or density field is then interpolated to define a value at each pixel across the image. Along each perpendicular axis, the spatial mean is subtracted from each value along that axis, yielding the fluctuations about the mean (middle). These fluctuations are then correlated along the perpendicular axis for each grid position over the image. Each experiment’s average velocity divergence is measured across the experiment’s run time to ensure that the system is not contracting or aging (right). Bars indicate ± standard deviation. (b) F-actin nematic director fluctuation autocorrelations (blue) exhibit the characteristic *k*_⊥_^-2^ scaling. The nematic director field is calculated from coarse-grained confocal images of a passive, in vitro F-actin network in the presence of 0.25% MC (default experimental conditions). As a control, the nematic director field is scrambled (orange) before calculating the autocorrelations, yielding a flat curve (~*k*_⊥_^0^). (c) Microtubule (MT) nematic director fluctuation autocorrelations (blue) within mitotic spindles, extracted from *Xenopus laevis* oocytes^19^, also follow the expected *k*_⊥_^-2^ scaling relationship, agreeing with previously published results^19^. The fluctuation autocorrelations are calculated using the same method described in this study (Supplementary Methods – Calculating Fluctuation Autocorrelations), where a nematic director field is generated from coarse-grained confocal images. Scrambling the data (orange) destroys this scaling behavior and flattens (~*k*_⊥_^0^) the autocorrelation sequence. (d) Nematic director fluctuation autocorrelations (blue) calculated using phase data from the *Xenopus laevis* oocyte mitotic spindle^19^, as described previously. The phase data was acquired using an LC-Polscope, which provides orientation information for every pixel in the image, and these orientation fluctuations exhibit the expected *k*_⊥_^-2^ scaling relationship, agreeing with previously reported scaling exponents^19^. Scrambled data (orange) exhibits a flat autocorrelation (~*k*_⊥_^0^) for all wavenumbers.


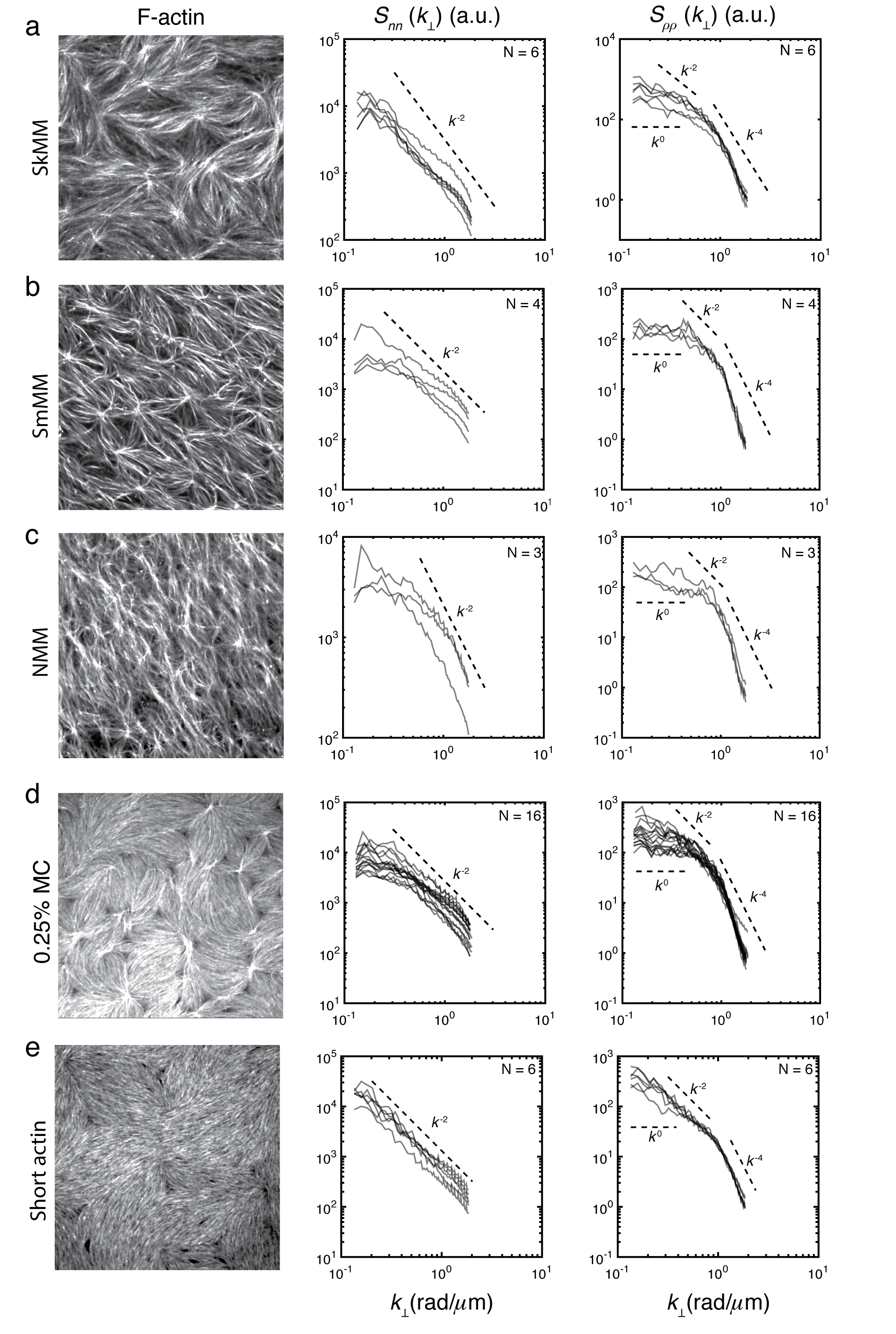


**Supplementary Figure 6 Fluctuation autocorrelation data for individual experiments.**

(a) Skeletal muscle myosin (SkMM, N= 6), (b) smooth muscle myosin (SmMM, N=4), (c) non-muscle myosin (NMM, N=3), (d) control (0.25% MC, N=16), and (e) short actin (N=6, 〈*l*〉 = 2.46 ± 0.86 μm, averaged over 200 filaments) experimental conditions analyzed to calculate mean fluctuation autocorrelations in the main text. Snapshot of a typical network (left column), the director-director autocorrelation function (middle column), and the density-density autocorrelation function (right column), as a time average for every experiment analyzed. Dashed lines in each plot indicate the expected scaling behavior due to active gel theory.


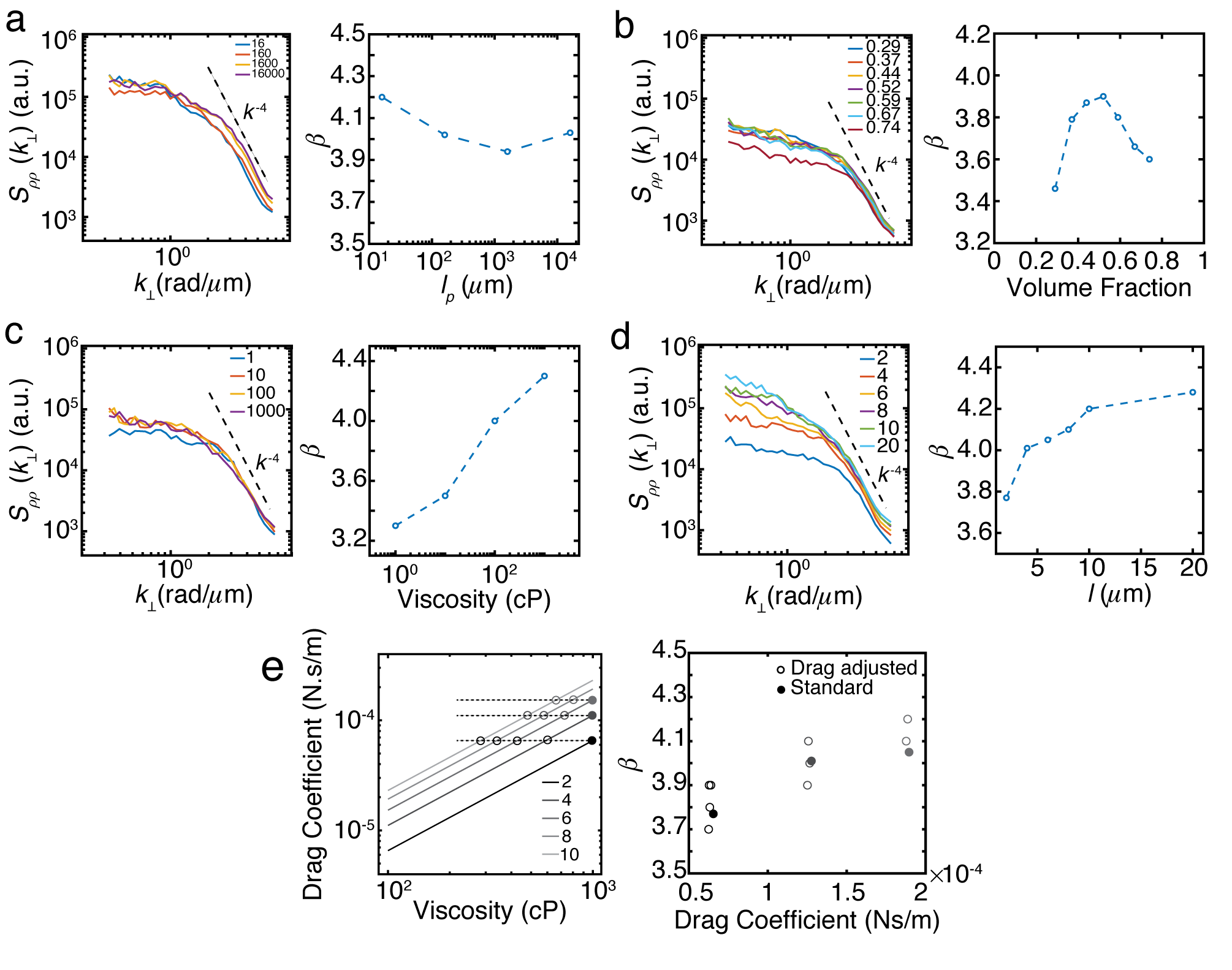


**Supplementary Figure 7 Parameter scan for agent-based simulations**

Effect of different simulation parameters on behavior of power law exponent *β*, quantifying the decay of density autocorrelation in the large *k*_⊥_ regime. (a) An increase in persistence length leads to a decay in *β*. (b) A change in volume fraction changes *β* non-monotonically. (c) The exponent *β* increases with simple viscosity *η*. (d) The strongest effect is observed in the case of changing length where *β* increases with an increase in length. This would imply a filament length-dependent drag as a controlling parameter for *β*. (e) To investigate this, we choose viscosities and filament lengths that give the same drag coefficient. For example, the simulation parameters given above use a viscosity of 1000 cP. By drawing a horizontal line for a filament of length 10 at 1000 cP (filled circle), we find the equivalent drag for filaments of different lengths (hollow circles). In the right column, we plot *β* as a function of both the original drag coefficients (filled circles) and for adjusted drag coefficients (hollow circles). We observe that *β* is similar for similar values of drag coefficients and increases as effective drag on filament increases.


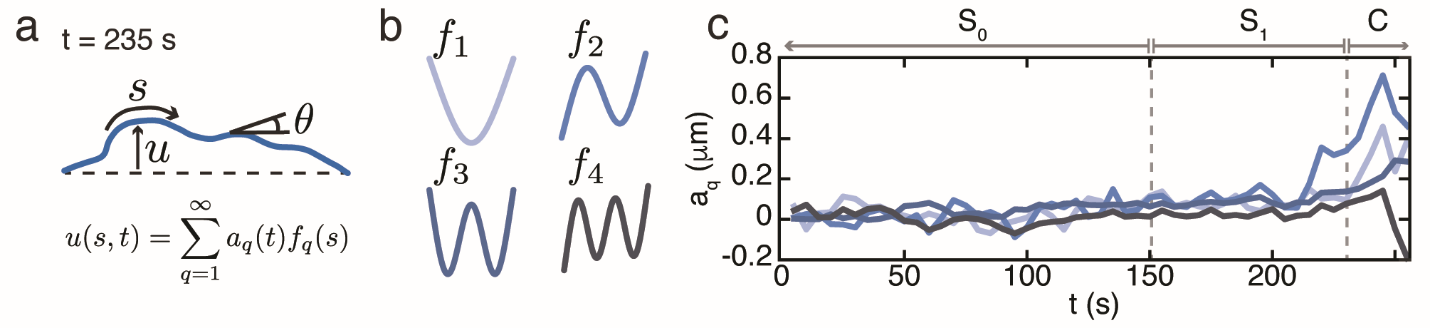


**Supplementary Figure 8 Filament normal mode analysis**

(a) Illustration of normal mode analysis done on filament traces at each time point. The tangent angle, *θ*, is found along the arc length *s* of the filament at each time *t*. These functions are then decomposed into a set of orthogonal normal modes, *f_q_*(*s*), whose coefficients *a_q_*(*t*) are tracked in time. (b) First four normal modes. (c) Time series of first four normal modes for the filament shown in Fig 4a in main text with corresponding states of actomyosin indicated by gray arrows.


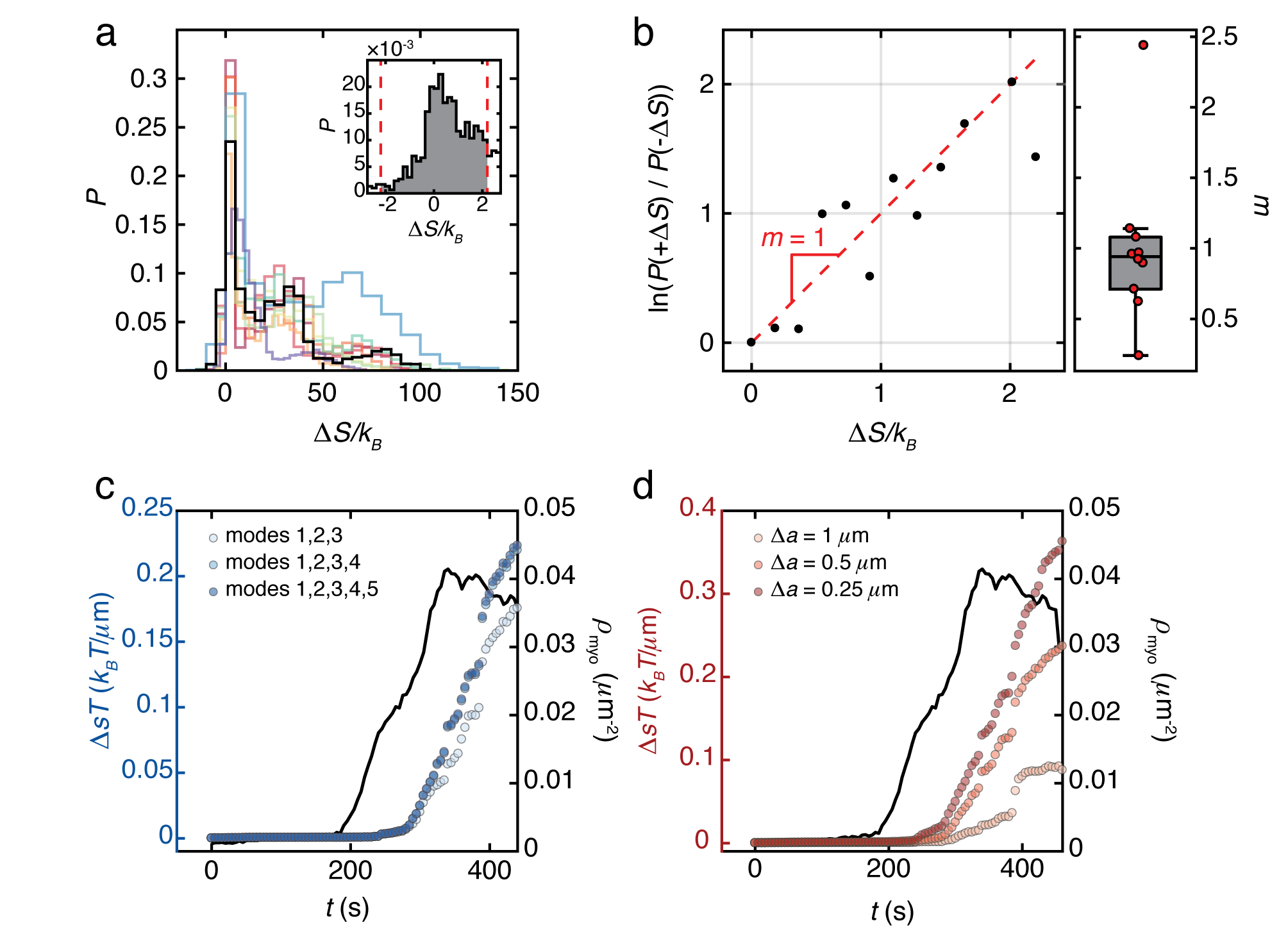


**Supplementary Figure 9 DFT and coarse-graining controls**

(a) Probability distribution function of entropy production values measured for 10 wild-type axonemes^16, 17^ using the same procedure as used for the actin filaments. Inset shows a narrow region around Δ*S* = 0 used to test the detailed fluctuation theorem (DFT) for the black distribution in the main figure. (b) Natural logarithm of the ratio of probabilities for positive and negative values of entropy in the region highlighted in gray in the inset of (a). Red dotted line shows the expected theoretical result, a line of slope 1. Box and whisker plot on the right shows the slopes of linear fits of DFT for all 10 axonemes shown in (a), showing most axonemes are clustered around a slope of 1. Box shows inter-quartile of data, whiskers show extent of the rest of the data, and black line inside of box shows median. (c) Effects on the measured entropy production when considering different numbers of bending modes. Including more modes slightly increases the measured entropy, but gives the same qualitative results. Points show average Δ*sT* across n=19 filaments. (d) Same as (c) but investigating the effects of the bin size used on the measured entropy production. While results are qualitatively the same as bin size varies, the dissipation energy magnitude increases with decreasing bin size.


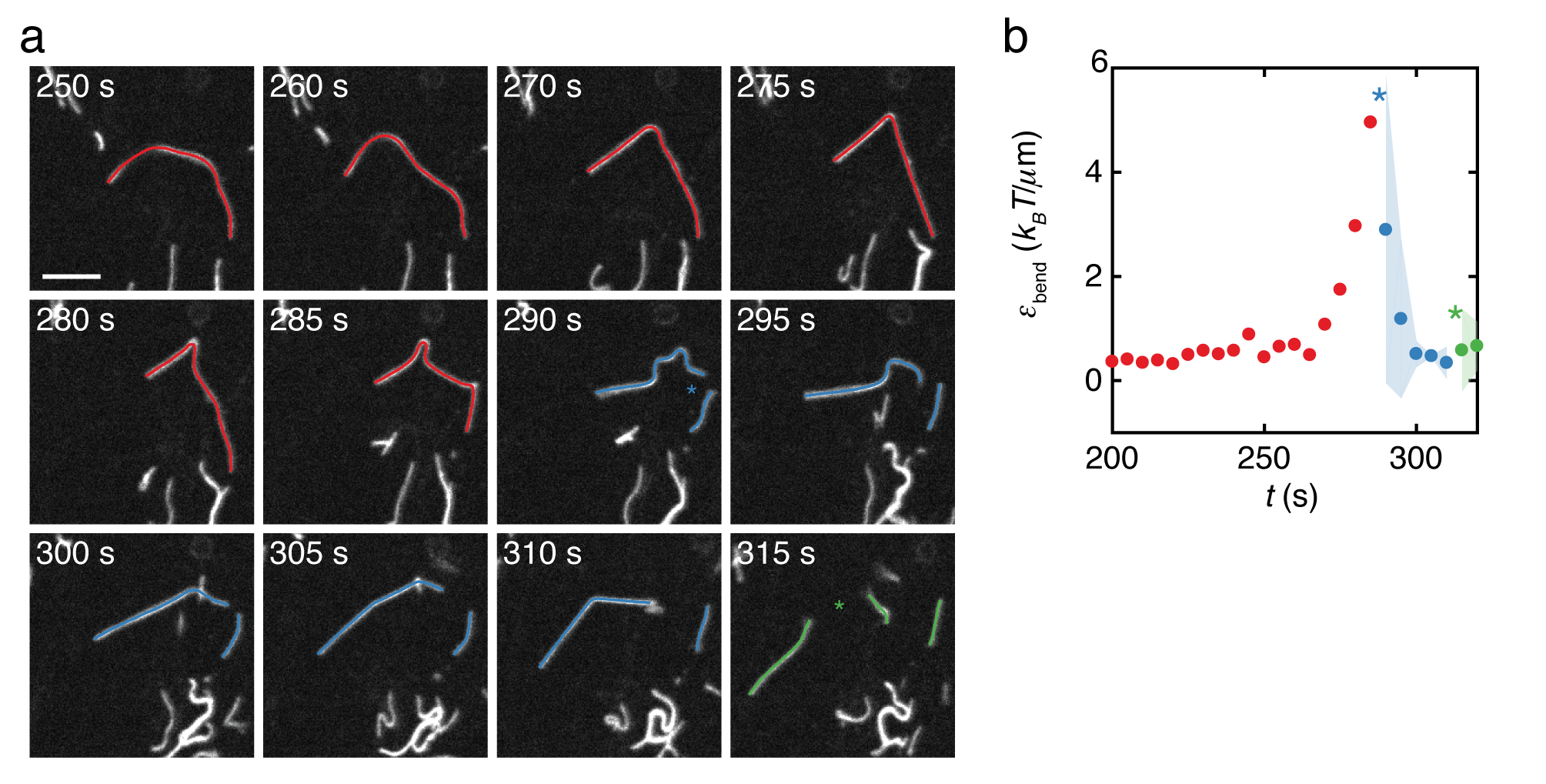


**Supplementary Figure 10: Filament severing decreases bending energy**

(a) Tracking a single filament in a sparsely labeled experiment as it severs. Filament shown in red leading up to first severing event, after which the filament is shown in blue. Blue asterisk shows location of first filament break. Filament is then shown in green after next severing event, with green asterisk showing location of the filament break. (b) Bending energy per unit length for the filaments traced in (a). Colors correspond to same periods of time as shown in (a). The bending energy density increases leading up the first severing event, indicated by the blue asterisk, after which is drops precipitously. Points and shaded area shows mean ± standard deviation of bending energies.

**Supplementary References**

1. Kohler S, Lieleg O, Bausch AR. Rheological characterization of the bundling transition in F-actin solutions induced by methylcellulose. *PloS one* **3**, e2736 (2008).

2. Cetera M*, et al.* Epithelial rotation promotes the global alignment of contractile actin bundles during Drosophila egg chamber elongation. *Nature communications* **5**, 5511 (2014).

3. Marchetti MC*, et al.* Hydrodynamics of soft active matter. *Rev Mod Phys* **85**, (2013).

4. Kruse K, Joanny JF, Julicher F, Prost J, Sekimoto K. Generic theory of active polar gels: a paradigm for cytoskeletal dynamics. *Eur Phys J E* **16**, 5-16 (2005).

5. Gennes PGd. *The physics of liquid crystals, by p. G. de Gennes*. Clarendon Press (1974).

6. Nedelec F, Foethke D. Collective Langevin dynamics of flexible cytoskeletal fibers. *New J Phys* **9**, (2007).

7. Onsager L. The Effects of Shape on the Interaction of Colloidal Particles. *Ann Ny Acad Sci* **51**, 627-659 (1949).

8. Doi M, Edwards SF. *The theory of polymer dynamics*. Clarendon Press ;

Oxford University Press (1986).

9. Smith MB, Li H, Shen T, Huang X, Yusuf E, Vavylonis D. Segmentation and tracking of cytoskeletal filaments using open active contours. *Cytoskeleton* **67**, 693-705 (2010).

10. Aragon SR, Pecora R. Dynamics of Wormlike Chains. *Macromolecules* **18**, 1868-1875 (1985).

11. Weiss JB. Coordinate invariance in stochastic dynamical systems. *Tellus A* **55**, 208-218 (2003).

12. Seifert U. Stochastic thermodynamics, fluctuation theorems and molecular machines. *Reports on progress in physics Physical Society* **75**, 126001 (2012).

13. Battle C*, et al.* Broken detailed balance at mesoscopic scales in active biological systems. *Science* **352**, 604-607 (2016).

14. Garcia de la Torre JG, Bloomfield VA. Hydrodynamic properties of complex, rigid, biological macromolecules: theory and applications. *Q Rev Biophys* **14**, 81-139 (1981).

15. Jarzynski C. Hamiltonian derivation of a detailed fluctuation theorem. *J Stat Phys* **98**, 77-102 (2000).

16. Sartori P, Geyer VF, Scholich A, Jülicher F, Howard J. Data from: Dynamic curvature regulation accounts for the symmetric and asymmetric beats of Chlamydomonas flagella. (ed^(eds). Dryad Data Repository (2016).

17. Sartori P, Geyer VF, Scholich A, Julicher F, Howard J. Dynamic curvature regulation accounts for the symmetric and asymmetric beats of Chlamydomonas flagella. *Elife* **5**, (2016).

18. Speck T, Blickle V, Bechinger C, Seifert U. Distribution of entropy production for a colloidal particle in a nonequilibrium steady state. *Epl-Europhys Lett* **79**, (2007).

19. Brugues J, Needleman D. Physical basis of spindle self-organization. *Proceedings of the National Academy of Sciences of the United States of America* **111**, 18496-18500 (2014).
