# Supplementary material for: Entropy production rate is maximized in non-contractile actomyosin
Source: Nat Commun. 2018 Nov 23;9:4948. doi: 10.1038/s41467-018-07413-5 (PMC6251913; doi:10.1038/s41467-018-07413-5)
Supplement: Supplementary file 9 — Description of Additional Supplementary Files [file 41467_2018_7413_MOESM9_ESM.pdf]

Titles and legends for Supplementary Movies are as follows:

- **Movie 1 Depletion Forces Induce Self-Organization of F-actin into 2D Nematically Ordered Domains**
  Fluorescently labeled actin (left), heatmap showing nematic order parameter field (middle) and spatially averaged nematic order parameter (right) over time while the quasi-2D biomimetic F-actin network organizes itself into nematic domains. Without adhesion to the underlying surface, the actin filaments crowd to the lipid bilayer on the coverslip (Fig 1a) due to the entropic depletion force of methylcellulose (MC), and entropic interactions between filaments rearrange the filaments into nematically ordered domains.
- **Movie 2 Nematic Order Decreases Prior to Network Strain in Contractile Networks**
  Fluorescently labeled actin (left), heatmap showing nematic order parameter field (middle) and spatially averaged nematic order parameter (right) over time as myosin-induced forces contract the network into aster-like formations. Decreases in the nematic order parameter (right, green) precede net contraction (right, blue), as measured by the average of the divergence of the velocity field of the actin measured by particle image velocimetry.
- **Movie 3 Nematic Order in a Non-Contractile Active Steady State**
  Fluorescently labeled actin (left), fluorescently labeled non-muscle myosin (NMM, middle), and heatmap showing nematic order parameter field (right) in a non-contractile steady state under the influence of myosin-induced active stresses. As myosin accumulates, the actin network undergoes dynamic restructuring, but macroscopic flow (Movie 2) is not seen. This steady state is also accompanied by a decrease in the nematic order parameter over space.
- **Movie 4 Simulation of Actomyosin Contractility**
  An agent based simulation of motor driven rearrangement of an actin network. The filaments initially grow in a quasi-3d volume to reach a predetermined length distribution and volume fraction. The filaments are color coded by length. Starting from t = 4.5 s to t = 14.5 s, the motor concentration is linearly increased to mimic deposition of myosin in experiments. The motors (blue dots) reorganize the initial distribution to an aster.

- **Movie 5 Entropy in Stable and Contractile States**
  The left panel in the movie shows filaments color coded by their instantaneous. The entropy values are scaled by the maximum entropy. On the right we plot the simultaneous temporal evolution of ensemble mean entropy along with the instantaneous divergence of the actin velocity field. A negative value of instantaneous divergence indicates a contractile state and vice versa.
- **Movie 6 Work in Stable and Contractile States**
  The left panel in the movie shows filaments color coded by their instantaneous bending energy. The bending energy values are scaled by the maximum bending energy. On the right we plot the simultaneous temporal evolution of ensemble mean bending energy along with the instantaneous divergence of the actin velocity field. A negative value of instantaneous divergence indicates a contractile state and vice versa.
- **Movie 7 Transverse Plucking of Actin Filaments by Non-Aligned Myosin**
  Fluorescently labeled actin (red) and smooth muscle myosin (SmMM, green) in a stable, actomyosin system. Time stamp is MM:SS. Individual instances of plucking motions can be seen (e.g. top right, 6-8 minute mark).
